# 4,000-year-old *Mycobacterium lepromatosis* genomes from Chile reveal long establishment of Hansen’s disease in the Americas

**DOI:** 10.1038/s41559-025-02771-y

**Published:** 2025-06-30

**Authors:** Darío A. Ramirez, T. Lesley Sitter, Sanni Översti, María José Herrera-Soto, Nicolás Pastor, Oscar Eduardo Fontana-Silva, Casey L. Kirkpatrick, José Castelleti-Dellepiane, Rodrigo Nores, Kirsten I. Bos

**Affiliations:** 1 Instituto de Antropología de Córdoba CONICET-UNC, Museo de Antropologías, Departamento de Antropología, Facultad de Filosofía y Humanidades, Universidad Nacional de Córdoba, Córdoba, Argentina; 2 Max Planck Institute for Evolutionary Anthropology, Leipzig, Germany; 3 Max Planck Institute of Geoanthropology, Jena, Germany; 4 Facultad de Filosofía y Letras, Universidad de Buenos Aires, Buenos Aires, Argentina; 5 Instituto de Antropología de Córdoba CONICET-UNC, Museo de Antropologías, Facultad de Filosofía y Humanidades, and Departamento de Diversidad Biológica y Ecología, Facultad de Ciencias Exactas, Físicas y Naturales, Universidad Nacional de Córdoba, Córdoba, Argentina; 6 Museo Arqueológico de La Serena, La Serena, Chile; 7 Department of Archaeology, Simon Fraser University, Burnaby, British Colombia Canada; 8 Department of Anthropology, Western University, London, Ontario Canada; 9 Independent researcher, Arica, Chile

**Keywords:** Microbial genetics, Biological anthropology

## Abstract

*Mycobacterium lepromatosis* is a recently identified cause of Hansen’s disease, and is associated with the more severe and potentially lethal presentations of diffuse lepromatous leprosy and Lucio’s phenomenon. Detection of this infection has been limited to a small number of individuals, leaving much to be learned about its global distribution and transmissibility. Its discovery in wild rodent populations in the United Kingdom and Ireland also raises questions about its zoonotic potential. Here, we raise further awareness of this disease via analyses of two exceptionally well preserved *M. lepromatosis* genomes obtained from 4,000-year-old human remains of two adult males from the archaeological sites of El Cerrito and La Herradura in Northern Chile. This formed the basis of genomic comparisons between ancient and modern forms of the pathogen. We demonstrate an unexpected long history of *M. lepromatosis* in the Americas, which contrasts with the more recent Eurasian history of the closely related *Mycobacterium leprae*. We offer relevant perspectives on its evolution while providing an incentive for further disease monitoring in both humans and other potential reservoir species in the Americas and elsewhere.

## Abstract (web summary)

Recovery of ancient *Mycobacterium lepromatosis* genomes in 4,000-year-old human remains from Chile demonstrates that this causal agent of Hansen’s disease has a long history in the Americas, predating the colonial period by millennia.

## Main

Hansen’s disease, more commonly known as leprosy, is caused by the unculturable bacteria *Mycobacterium leprae* and the recently described *Mycobacterium lepromatosis*^1^. Transmission occurs via prolonged exposure to respiratory droplets from an infected person^2^. Untreated individuals can develop a chronic peripheral neuropathy with associated physical impairment^3^. Many infected remain asymptomatic, which can obscure diagnoses and control measures^4^. The availability of curative multidrug treatments has decreased worldwide prevalence^5^; regardless, the disease persists in more than 100 countries, with up to 174,000 new cases reported globally in 2022 alone^6^. Risk of infection is closely correlated with conditions of overcrowding, poverty, malnutrition and an immunocompromised state^7^.

Written accounts describe the impact of disfiguring diseases presumed to be Hansen’s disease on Eurasian populations throughout the historic period^8^. As skeletal involvement occurs in advanced stages^9^, past infections have been identified in archaeological tissues as early as 5,000 years ago in Europe, Asia and Oceania^10–15^. For *M. leprae*, analyses of ancient genomic data provide further support for its infectious potential having spanned several millennia^16^. While humans are regarded as the principal host of Hansen’s disease, maintenance of the causative bacteria in other animal species has raised concerns over their potential as zoonotic reservoirs from a One Health perspective^17^. Nine-banded armadillos are known sources of *M. leprae*, where transmission may occur through human consumption^18^. Red squirrels in Britain and Ireland can harbour both *M. leprae* and *M. lepromatosis*^19^, and recent identification of *M. leprae* in archaeological rodent bone demonstrates cross-species infectivity in historical periods^20^. Detection of *M. leprae* in several species of non-human primates further demonstrates the broad host range of this pathogen^21–23^. Viability of *M. leprae* in ticks and amoebae for several months opens the possibility of environmental reservoirs as well^24,25^. Unlike many bacterial diseases, presentation of symptoms and the development of its more severe multibacillary or lepromatous forms seem highly dependent on host immunological status^2,26^. While the few available reports tend to associate *M. lepromatosis* with severe disease presentation such as diffuse lepromatous leprosy (DLL) and the potentially fatal Lucio’s phenomenon (LP), a set of clinically defined criteria that distinguish it from *M. leprae* infection has yet to be established^27^.

Understanding of *M. lepromatosis* distribution and evolutionary history is limited as few examples of the infection have been molecularly confirmed. Polymerase chain reaction (PCR)-based detections demonstrate its presence in the Americas (Mexico and the Caribbean)^1,28^, as well as Southeast Asia (Myanmar and Singapore)^29^, consistent with the global occurrence of DLL^27,30^. Genome-level analyses are limited in scope: the available modern genomes suggest a deep divergence of *M. lepromatosis* and *M. leprae*, although with retention of genomic features that contribute to some similarities in disease presentation^31^. While investigations that draw upon both modern and ancient genomic data consistently support an origin for *M. leprae* outside the Americas^16^, the identification of *M. lepromatosis* in archaeological contexts has not been reported, although its modern association with Latin American contexts has led to the hypothesis of its endemicity in the continent in the precolonial period^30,31^.

Palaeogenomic investigations of this disease are currently restricted to the recovery of *M. leprae* genomes and are dominated by studies that are limited to a Eurasian context. Here, we present two high-coverage *M. lepromatosis* genomes reconstructed from skeletal remains of individuals from distinct archaeological contexts from Chile, both dated to about 4,000 years ago. These data indicate a long and previously undocumented history of this infectious disease in the Americas.

## Archaeological context, morphology and molecular recovery

**Fig. 1:**
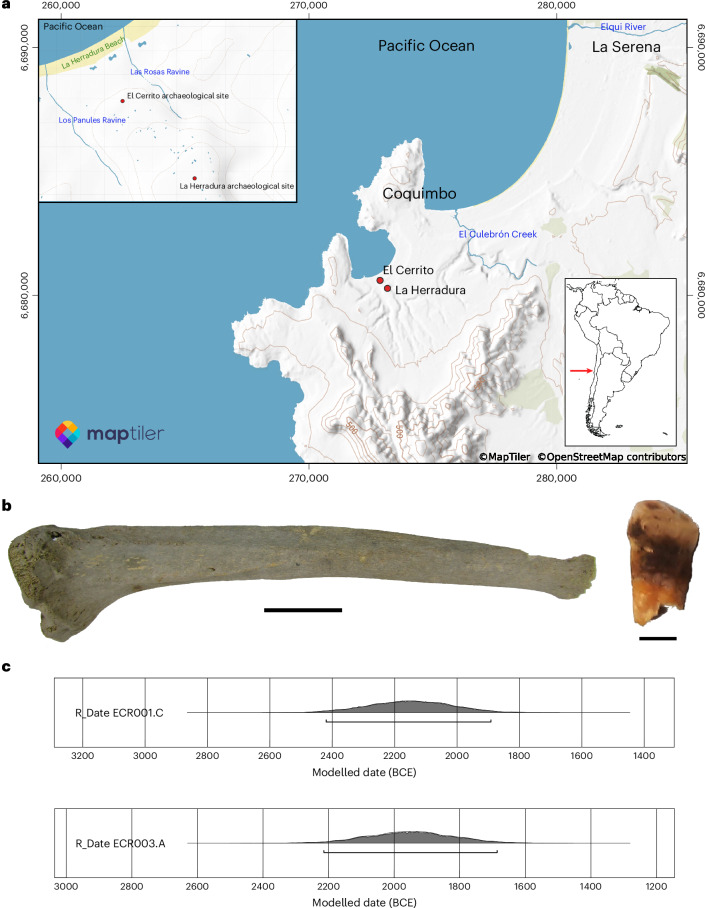
Archaeological context for the skeletal elements that yielded ancient *M. lepromatosis* DNA. **a**, Map of the semi-arid region of Chile showing the location of the two archaeological sites under study. Coordinates follow the universal transverse mercator (UTM) system (Datum WGS 84, Zone 19J); values are given as easting and northing (m). Map created with the MapTiler plugin within QGIS. **b**, Skeletal elements that yielded the two ancient genomes of *M. lepromatosis*: left, tibia from ECR001 (scale bar, 5 cm); right, tooth from ECR003 (scale bar, 0.5 cm). **c**, Modelled radiocarbon dates of the individuals ECR001 and ECR003 from La Herradura and El Cerrito sites, respectively. Basemap © MapTiler and OpenSteetMap contributors. Inset map of South America from pyty/Depositphotos.com. Panel **c** created with OxCal v.4.4.4 (ref. ^76^); radiocarbon dates calibrated using the Marine20 calibration curve^77^, with marine reservoir data from ref. ^78^.

To investigate infectious disease in the American precolonial period from a molecular perspective, we sampled 35 teeth and 19 bones with pathological lesions suggestive of active infection belonging to 41 individuals from five archaeological sites representing various time periods and subsistence strategies in the semi-arid region of Chile (Supplementary Information Section 1). Both teeth and pathological bone were selected to permit identification of pathogens that contribute to either acute or chronic infection, and when available both tissue types were selected from an individual. Approximately 50 mg of each tissue was extracted and converted into a single-stranded DNA library for sequencing on an Illumina HiSeq 4000 to a depth of ~5 million reads. Data were computationally screened for a variety of pathogenic bacteria and viruses following an hypothesis-free method using the MALT and HOPS platforms implemented through the nf-core EAGER 2 analysis pipeline^32–34^. This process revealed several thousand DNA fragments with homology to *M. lepromatosis* in each of two archaeological tissues, representing the neighbouring sites of La Herradura (a tibia from an individual referred to here as ECR001) and El Cerrito (a tooth from an individual referred to here as ECR003) (Fig. 1, Supplementary Tables 1 and 2 and Supplementary Figs. 1–6). Radiocarbon dating of both skeletal elements indicate them to be roughly contemporaneous, from around 3,900–4,100 calibrated years ago (Fig. 1).

Currently there is little information on the osteological manifestations of *M. lepromatosis* infection, but most reported examples are associated with the DLL and LP forms of Hansen’s disease^1^. DLL primarily affects the skin and peripheral nerves but it can also cause ocular damage, rhinitis, destruction of the nasal septum causing saddle or crooked nose (usually without affecting the nasal bones), damage to the larynx, organ damage or failure and sepsis. Generalized hypaesthesia or anaesthesia resulting from neuritis can contribute to secondary injury of the extremities, which may result in bony changes. LP is a rare reaction most commonly associated with DLL that manifests as acute, necrotizing cutaneous vasculitis, generally affecting the legs, arms, torso and face^35^. Although LP does not necessarily affect the bones, the resulting inflammation and possible secondary infections could potentially cause osteological changes. Genetically confirmed *M. lepromatosis* infections have also been associated with borderline lepromatous leprosy and lepromatous leprosy^27^, the latter being the most common form of Hansen’s disease to cause osteological changes^36^. While its modern presentation may differ from the spectrum of pathology observed in the past, both individuals display pathological lesions that are consistent with, although not diagnostic of Hansen’s disease, as well as additional changes that are not typically associated with Hansen’s disease and may be a result of unrelated afflictions (see Supplementary Information for complete descriptions of the remains). For example, skeleton ECR001 (male 35–40 years, Supplementary Figs. 2–4) exhibits a slight widening of the nasal aperture compared to other individuals in the population, with rounding of the margins and possible osteolytic processes in the area. This individual also has slight recession of the alveolar bone of the anterior teeth (although this may be in part due to taphonomic breakage or in response to other pathological processes), as well as pitting on the palatine process and on the ribs. The right fibula and tibia are affected by mostly healed lamellar periostosis and slight thickening and bowing of the right tibial diaphysis. The small tubular bones of the hands display pitting, abnormal foramina and periosteal new bone on the palmar surfaces, but no concentric resorption or evidence of hyperflexion, and there are pronounced osteolytic lesions on the right calcaneus. Skeleton ECR003 (male, 40–44 years) has fewer preserved skeletal elements but also displays rounding of the inferior margins of the nasal aperture and slight thickening and bowing of the tibial diaphysis (Fig. 1 and Supplementary Fig. 5). Although the aforementioned osteological changes in both individuals could be associated with Hansen’s disease (though not necessarily with the DLL or LP forms), they could equally be caused by other diseases, both infectious and non-infectious. For this reason, we do not attempt a differential diagnosis based on osteological criteria, nor do we propose any new diagnostic criteria from these limited examples.

To explore the suitability of genomic reconstruction, DNA libraries were enriched via in-solution capture through use of a probe set designed from a modern *M. leprae* reference panel^37^, and sequenced to a read depth of 20 million fragments, as above. Distinction between several mycobacterial species was accomplished via a competitive mapping approach, which demonstrated much higher homology and hence high confidence in their assignment to *M. lepromatosis* (Supplementary Table 3). Both genomes are of exceptional quality, yielding average genomic coverages of 45- and 74-fold for ECR001 and ECR003, respectively, when mapped against the modern FJ924 *M. lepromatosis* genomic reference (CP083405) (Supplementary Table 4), isolated from a patient in Mexico^38^. The distribution of heterozygous positions is consistent with a single source of *M. lepromatosis* DNA for each individual, although with a detectable level of chemical damage and possibly sporadic reads of non-target origin in the mapped datasets (Supplementary Fig. 7), as expected of metagenomically sourced ancient bacterial DNA. The spectrum of DNA damage from both pathogen and host (Supplementary Figs. 6 and 8) is consistent with their contemporaneous antiquity as determined from radiocarbon data (Fig. 1). An analysis of human DNA also indicates an exclusively American Indigenous host source (Supplementary Table 5). Negative controls were free of *M. lepromatosis* DNA (Supplementary Table 6).

## *M. lepromatosis* pangenome and comparisons against *M. leprae*

**Fig. 2:**
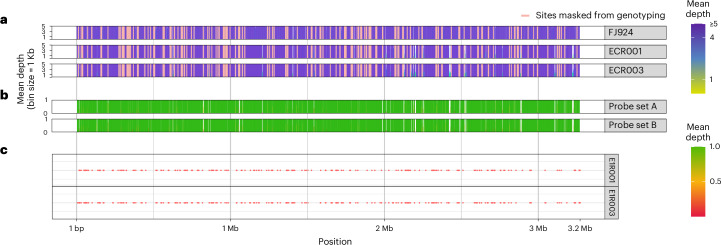
Overview of the recovery status of the newly identified ancient *M. lepromatosis* genomes. **a**, Indication of the genomic regions with a depth range between zero and fivefold averaged over bins of 1,000 bp. The top panel depicts an artificially fragmented dataset of the reference genome FJ924 mapped to itself to identify regions of low mappability. **b**, Genome coverage of the *M. leprae* probes mapped to the *M. lepromatosis* FJ924 reference genome averaged over bins of 1,000 bp (top and bottom represent two different probe sets that are pooled for enrichment). **c**, Visual representation of the location of the non-reference loci recovered for ECR001 and ECR003.

**Extended Data Fig. 1:**
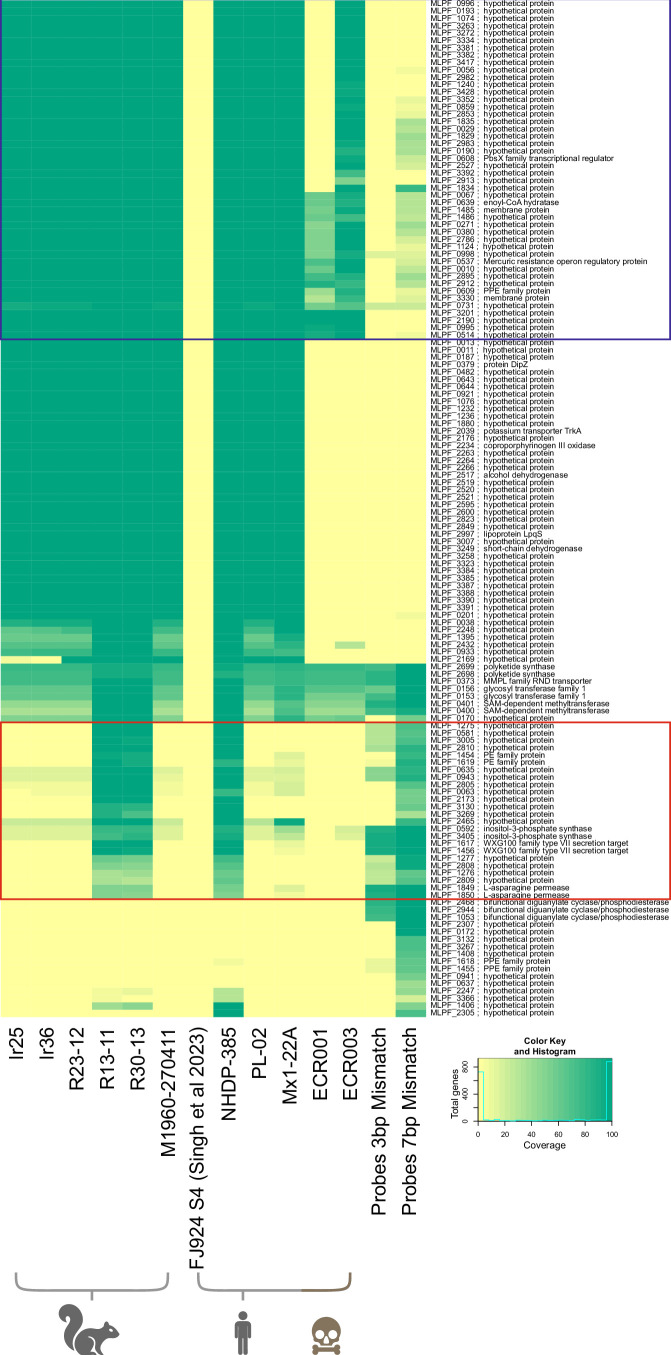
Heat map of relative mapping coverage over annotated coding regions of the FJ924 *M. lepromatosis* genome. Plot is restricted to all annotated genes that have less than 85% coverage at 4-fold read depth in any of the genomes or less than 85% average coverage at 1-fold depth in the probe set at the stated mapping stringency (based on a pooled dataset of ‘A’ and ‘B’ probes). Red square indicates genes of interest with asymmetric coverage across species-associated lineages. Blue box indicates genes with coverage in either ancient genome where coverage in the probes is low.

Despite our use of an *M. leprae* capture panel, we observed a 279- and 23-fold increase in *M. lepromatosis* DNA content between the shotgun and enriched datasets, with 83% and 88% of the *M. lepromatosis* genome covered at fourfold read support for genomes ECR001 and ECR003, respectively (Fig. 2 and Supplementary Tables 3 and 4). To investigate possible enrichment biases over individual regions, probes were mapped with high sensitivity against the *M. lepromatosis* reference and probe coverage was compared to that observed in the two ancient genomes over annotated coding regions (Fig. 2, Supplementary Fig. 9 and Extended Data Fig. 1). Both ancient genomes include coverage over regions of the *M. lepromatosis* reference that were not included in probe design and hence were not enriched. Coverage across these regions is higher for genome ECR003, which may be due to a higher abundance of *M. lepromatosis* DNA in the non-enriched fraction (Supplementary Table 3). Importantly, we identify several regions with limited mapping reads in both ancient genomes where probe coverage is abundant. Further investigation revealed these regions to have asymmetric representation across host-associated modern genomes, which could indicate lineage-specific losses unrelated to host adaptation. This also reveals no pattern of gene loss/acquisition that distinguishes ancient from modern forms (Extended Data Fig. 1).

This analysis was complemented by evaluation of the two reconstructed ancient genomes alongside 16 modern *M. lepromatosis* (Supplementary Table 7) based on a common mapping to the FJ924 reference (Supplementary Table 8 and Supplementary Figs. 10 and 11). No consistent pattern of gene acquisition or loss across the full annotated coding region distinguishes the human-associated strains from those associated with red squirrel populations in the north of the United Kingdom or Ireland (Supplementary Fig. 11). This implies that any long-term changes related to host-specificity are influenced by either nucleotide substitution, disruptions in synteny or changes outside of the mapped coding regions that are undetected via the methods used here. This analysis also revealed a surprisingly low coverage for genome FJ924_S_4 reported in ref. ^39^ as a first example of *M. lepromatosis* in India (Supplementary Table 9 and Supplementary Figs. 10 and 11). A competitive mapping approach revealed this genome to show far greater homology to *M. leprae*, thus questioning the accuracy of its assignment to *M. lepromatosis* (Supplementary Table 9).

Given the established observation of genome decay and reduction in *M. leprae* over evolutionary timescales^40^, divergence between *M. lepromatosis* and *M. leprae* was investigated on a gene level. There are currently four chromosomally resolved modern *M. leprae* genomes available, representative of branches 1 (*n* = 2), 3 (*n* = 1) and 4 (*n* = 1). A pangenomic analysis carried out in Roary^41^ indicated a strong level of divergence between the two pathogens, with 2,000 (about half) of the 4,097 protein coding regions identified in Prokka showing a minimum of 50% sequence homology between the two pathogens (Supplementary Fig. 12). This demonstrates a high sequence divergence despite *M. leprae* having been identified as the most closely related organism to *M. lepromatosis*^31^. This is further demonstrated via a mapping-based approach, which reveals the two to share only ~25% nucleotide identity (Supplementary Table 10). An alignment of the genomes using LASTZ^42^ and MAUVE^43^ shows several large rearrangements and ~0.5 mega base pairs (Mb) (~12% of the genome) present in *M. lepromatosis* FJ924 that is absent in *M. leprae* MRHRU-235-G, either through acquisition in the former, decay in the latter or a nucleotide homology that is too low for alignment. Less similarity is observed with the more distantly related *Mycobacterium haemophilum* (Supplementary Figs. 12–14). This would leave only disparate regions of similarity upon which to perform downstream genome-level analyses where *M. leprae* or another mycobacterial representative are included.

## Phylogenetic analysis

**Fig. 3:**
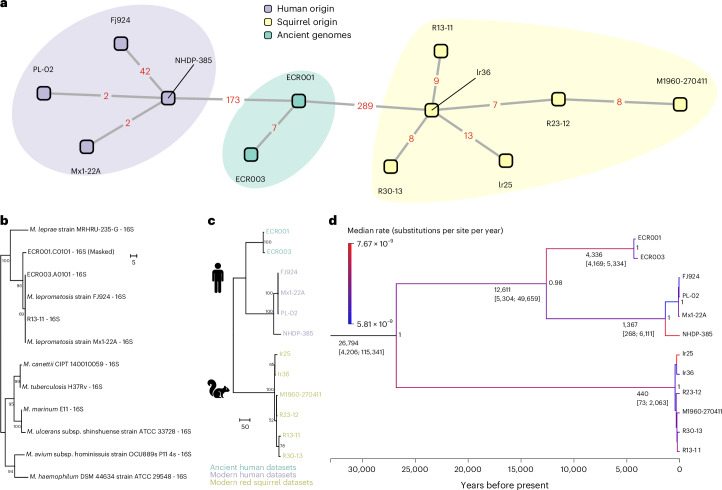
Phylogenetic representation of ancient and modern *M. lepromatosis.* **a**, Distance network generated using MEGA11 v.11.0.13 on the full SNP set (650 SNPs). The network is based on an unrooted UPGMA tree with a precomputed pairwise distance model set to ‘number of differences’ to calculate genetic distance in SNPs. Missing data were removed via ‘pairwise deletion’, which removed pairs where one dataset had an ambiguous character. This reduced the number of SNPs displayed in the network to 590. No bootstraps were needed. **b**,**c**, Maximum parsimony trees (with branch-length estimation) constructed in MEGA X v.11.0.13 (ref. ^45^) with 1,000 bootstrap iterations based on a 16S ribosomal RNA alignment of several mycobacterial representatives with ambiguous sites masked in the lower coverage genome ECR001 (**b**) and 650 full genomic alleles called at fourfold read support (**c**). **d**, Maximum clade credibility tree with median heights, reconstructed using the BSP and relaxed clock. Branches in **d** are colour-coded on the basis of the median rate estimates from the optimized relaxed clock model, with blue indicating lower rates and red indicating higher rates. Node labels show posterior support values.

The relationship of *M. lepromatosis* to other pathogenic mycobacteria was first determined through investigation of the 16S ribosomal RNA locus (Fig. 3b), which confirmed *M. leprae* to be its closest relative despite extensive genomic divergence described above. This was complemented by a conservative approach to genome-level phylogenetic reconstruction, where focus was restricted to diversity within *M. lepromatosis*. These data are limited to the two ancient genomes presented here, four modern human genomes from Mexico and six modern genomes isolated from red squirrels in Ireland and the United Kingdom. Single nucleotide polymorphisms (SNPs) were called at fourfold read support, and regions of low complexity, along with additional regions identified as potentially drawing background signal from co-enriched metagenomic DNA, were removed (Supplementary Table 11). While *M. leprae* has not been observed to undergo recombination, Gubbins^44^ was applied to investigate this phenomenon in this sparsely studied organism (Supplementary Table 11). These various filters resulted in 650 variant positions upon which to base the phylogeny (Fig. 3a and Supplementary Tables 12 and 13). A maximum parsimony tree was generated in MEGA11 (ref. ^45^) with 100 bootstraps, midpoint rooting and branch-length estimation (Fig. 3c). The phylogeny supports a robust separation between the human and rodent-associated lineages, where the two ancient genomes form a sister clade to the cluster of all human *M. lepromatosis* thus far sequenced at the genomic level. For all polymorphic positions, 94 occur uniquely in the ancient genomes of which 43 correspond to non-synonymous changes with potential functional significance (Supplementary Table 12).

## Emergence scenarios for *M. lepromatosis*

Reconstruction of the first ancient *M. lepromatosis* genomes with such deep chronology offers an unprecedented opportunity to explore the evolutionary history of the species. Using the radiocarbon ages of skeletal elements from ECR001 and ECR003 and the collection year for all modern genomes (Supplementary Table 14), time-calibrated phylogenetic trees were constructed to estimate divergence times and evolutionary rates using the BEAST v.2.7.7 software package^46^. Topology of the Bayesian phylogeny agrees with that inferred from parsimony (Fig. 3d). For thorough comparison we considered both strict and optimized uncorrelated relaxed log-normal clock models^47^ along with both the Bayesian skyline plot (BSP)^48^ and the coalescent constant population size model for demographic inference (Supplementary Information Section 5.1). Model selection strongly supported a relaxed clock with BSP indicating rate heterogeneity among branches (Supplementary Table 15a), which may reflect host-specific adaptations within human- and rodent-associated lineages.

Strength of temporal signal in the data was investigated via date randomization test^49^ (Supplementary Figs. 15 and 16). Simulations here showed a small proportion of overlap in the clock rate parameter (Supplementary Fig. 17), which indicated that a Bayesian framework may not estimate evolutionary rates and timescales with high confidence. This limitation probably arises from the small number of available genomes. We, therefore, chose to apply a prior distribution for the rate parameter based on previous estimates inferred for *M. leprae* (Supplementary Information Section 6.3). The best-supported model estimates an evolutionary rate of 6.91 × 10^−9^ substitutions per site per year (95% HDPI: 0.34 × 10^−9^–15.64 × 10^−9^ substitutions per site per year) for *M. lepromatosis*, which agrees closely with estimates obtained via other models (Supplementary Table 15b), as well as previous estimates for *M. leprae* genomic substitution rates (Supplementary Table 16). From this, we estimate the median time for the most recent common ancestor (tMRCA) of *M. lepromatosis* to be ~26,800 years ago (95% HPDI range of 4,206 to 115,340 yr bp) (Supplementary Table 15b). Genomes obtained from human hosts yield a divergence estimate of ~12,600 years (95% HPDI: 5,304 to 49,659 yr bp), while the tMRCA for the red squirrel clade is ~440 years (95% HPDI: 73 to 2,063 yr bp) (Supplementary Table 15b). The estimates proposed here are consistent with results obtained from all iterations tested, supporting robustness across different demographic and molecular clock models (Supplementary Table 15b and Supplementary Figs. 17–20). Our tMRCA for *M. lepromatosis* closely aligns with results presented elsewhere based on modern data^19^, although with broader temporal intervals resulting from either our inclusion of ancient genomes or our selection of more permissive models. Further refinement of the origin, evolution and relationship between the ancient strains and those from the regions where the disease is found today is expected to come with additional genomic examples made available through increased awareness for its detection in both clinical and archaeological contexts.

Recent investigations of *M. leprae*, as well as several other bacterial pathogens where ancient genomes are available, place their extrapolated coalescence date in the last 6,000 years, which correlates with cultural adaptations such as the adoption of agriculture and animal husbandry in the Neolithic that are regarded as conducive to the emergence and maintenance of new pathogens in human groups^16,50–53^. The current analysis reveals a different evolutionary history for *M. lepromatosis*: although based on only a few genomes, multiple simulations suggest a common ancestor for the human-associated lineages which temporally aligns with the Pleistocene–Holocene transition. This encompasses a warming period wherein human movements were less impeded by large ice sheets that covered 25% of the Earth’s land surface during the Last Glacial Maximum. Further exploration of the vast territories of the American continent soon followed, as demonstrated by the sudden increase in archaeological sites which indicate human activity^54^. This opens opportunities for acquisition of new infectious diseases and their transmission between connected groups. Our finding of two *M. lepromatosis* infections in South America, before the periods of known contact with either Oceanian or European populations, implies either movement of the pathogen within human groups during an early peopling event or its previously established endemicity in the continent in a separate reservoir species eventually acquired by humans. The latter would imply that its current distribution arises from a postcolonial dissemination, and would make it one of the few global diseases known to have emerged in the Americas^55^. Its presence in the continent has thus far remained undetected based on morphological analyses of human archaeological tissues, where skeletal lesions ascribed to Hansen’s disease are limited to examples from the postcolonial period^56^, with the exception of two potential infections from the northern Pacific Coast that await molecular characterization and confirmation of their possible pre-ad 1492 status^57^. Additional ancient genomes from either human or faunal remains may eventually disentangle the current mystery of its origin and possible means of acquisition among the hunting-gathering-fishing groups studied here. It may also assist in the establishment of morphological diagnostic criteria for disease identification in the archaeological record.

While we observe a deep divergence between the human- and rodent-associated lineages, current data from non-human sources are limited to modern rodent lineages within a restrictive geographic spread in Ireland and the United Kingdom^58^, from a single introductory event of unknown origin within the last 500 years. While surveillance has as yet failed to identify *M. lepromatosis* or *M. leprae* in several squirrel species in mainland Europe^59^, analogous efforts in other parts of the world are needed to explore its ecological distribution in broader scale. Greater awareness of this pathogen and its potential for zoonotic transmission from armadillos is also being explored given that they are known reservoirs of *M. leprae* in the Americas. Previous contact with these animals (handling or consumption) has been reported in two individuals with confirmed *M. lepromatosis* infection in Mexico^60^. Screening efforts of several species of armadillos have also begun in Brazil, where human infections with *M. lepromatosis* represent >10% of reported instances of Hansen’s disease^61^. Of note, both individuals studied here come from archaeological contexts in Chile that are outside the current range of armadillos.

## Modern *M. lepromatosis* in perspective

Since its discovery in 2008, *M. lepromatosis* has been regarded as a second causal pathogen for Hansen’s disease. While associated with the more severe forms of DLL and LP, these presentations are equally considered within the clinical spectrum of *M. leprae* infection^27^. Distinction between the two pathogens through use of the recently validated species-specific PCR assay^62^ has the potential to elucidate the true global prevalence of *M. lepromatosis*. Here, we aim to raise awareness of *M. lepromatosis* infection through demonstration of its previously unknown health impact along the Pacific Coast of South America several millennia in the past. This region currently has a low incidence of Hansen’s disease where occasional reported cases, thus far attributed to *M. leprae*, are thought to result from travel to regions within Latin America where disease incidence is high^63,64^. Its restricted modern geographic distribution may in part be due to its decreased transmissibility in comparison to other globally dispersed pathogens. Management of human infections in living populations remains a principal concern, and adoption of a One Health perspective could provide the means to elucidate the zoonotic potential of this disease both in the present as well as the past^20^. Available data suggest that squirrel populations in Britain and Ireland may be the sole non-human reservoir for these pathogens in West Eurasia^58,59^. The results of such screenings from rodent populations in East Eurasia have yet to be reported, and recent evidence suggests that wild rodents may be a natural source of *M. leprae* in Brazil^65^. This highlights the need for broader-scale investigations into potential wild reservoirs for both *M. leprae* and *M. lepromatosis*. The capacity of armadillos to harbour *M. lepromatosis* infection in Latin American countries, especially those where DLL representation is high, such as Mexico and the Caribbean^27^, should also be considered. Given the narrow known host range for *M. leprae*, susceptibility in rodents, armadillos or other animals may be related to their possible maintenance of *M. lepromatosis* in the past. Further contributions are also expected to come from palaeogenomic analyses that continue to explore past disease landscapes represented in both human and animal remains.

## Methods

Skeletal elements were selected for analysis on the basis of gross examination of morphological features suggestive of active infectious disease. See Supplementary Information Section 2 for a detailed description of the two individuals who yielded the *M. lepromatosis* genomes. Bone powders underwent a demineralization and protein-mediated lysis via 16-h incubation at 37 °C with 0.45 M EDTA buffer (pH 8.0, Thermo Fisher), 0.25 mg ml^−1^ of proteinase K (Sigma-Aldrich) and 0.05% Tween (Sigma-Aldrich) in Lo-Bind 2-ml Eppendorf tubes. Single-stranded DNA libraries were constructed from 400 µl of digested bone through an automated process, as described^66^, and were sequenced on an Illumina HiSeq 4000 using single-end 75 base pair (bp) chemistry to a depth of 5 million reads. Data were processed with the HOPS (Heuristic Operations for Pathogen Screening) pipeline^33^, which performs a metagenomic evaluation through MALT on the basis of a reference database of 6,000 full genomes from bacteria and viruses^32^, followed by extraction of reads assigned to a customized list of 1,915 reference pathogen genomes, including bacteria, virus, fungi and parasites. HOPS was run using nf-core/eager v.2.4.3 pipeline^34,67^. Sample and control libraries were enriched for *M. leprae* or *M. lepromatosis* DNA in two rounds of consecutive in-solution capture^68^ automated on the Bravo NGS workstation. Both non-enriched and enriched library products were sequenced on an Illumina HiSeq 4000 using single-end 75-bp chemistry. Damage patterns suggestive of ancient DNA were estimated by DamageProfiler v.0.4.9 (ref. ^69^) as implemented in nf-core/eager v.2.4.3 pipeline. Damage plots generated by the software were visually inspected and validate the antiquity of both human and pathogen DNA.

To evaluate human DNA content, shotgun and enriched data were analysed using nf-core/eager v.2.4.4 (ref. ^34^) (Supplementary Table 5). Adaptor sequences were trimmed with AdapterRemoval v.2.3.2 (ref. ^70^) using default parameters for removing low-quality reads (minimum read length = 30 and minimum base quality = 20). Before being merged, three bases were trimmed from both ends of each read using fastp v.0.20.1 (ref. ^71^). As DNA recovery in both individuals was too low for whole-genomic evaluation of ancestry, sequences were subsequently mapped to the revised Cambridge reference sequence (NC_012920.1) using bwa v.0.7.17 (ref. ^72^) with --bwaalnn 0.01 and --bwaalnl 32 for mitochondrial haplogroup determination. Mapped sequences were quality filtered using samtools v.1.12 (ref. ^73^) for a minimum quality of 37 and a minimum length of 30. VCF files were constructed using GATKs unified genotyper v.3.5 (ref. ^74^) resulting in a mean coverage of 3.6× and 2.4× for ECR001 and ECR003, respectively. HaploGrep v.2.4 (ref. ^75^) was used to assign mitochondrial DNA haplogroups, which were later manually confirmed. The resulting haplogroups indicated a Native American origin for both individuals: ECR001 belongs to A2 + (64) and ECR003 to D4h3a1a.

Additional methods regarding the genomic analyses and molecular dating can be found in Supplementary Information Sections 5 and 6, respectively.

### Reporting summary

Further information on research design is available in the Nature Portfolio Reporting Summary linked to this article.

## Supplementary information

Supplementary Information Supplementary Sections 1–6, Figs. 1–20 and references.

Reporting Summary

Supplementary Tables Supplementary Tables 1–15.

## Data Availability

Data are accessible via the European Nucleotide Archive (ENA) project ID ERR13916540 and ERR13916541.
