# Supplementary material for: 4,000-year-old Mycobacterium lepromatosis genomes from Chile reveal long establishment of Hansen’s disease in the Americas
Source: Nat Ecol Evol. 2025 Jun 30;9(9):1685–93. doi: 10.1038/s41559-025-02771-y (PMC12420385; doi:10.1038/s41559-025-02771-y)
Supplement: Supplementary file 1 — Supplementary Sections 1–6, Figs. 1–20 and references. [file 41559_2025_2771_MOESM1_ESM.pdf]

nature ecology & evolution

Supplementary information https://doi.org/10.1038/s41559-025-02771-y

4,000-year-old *Mycobacterium leprromatosis* genomes from Chile reveal long establishment of Hansen's disease in the Americas

In the format provided by the authors and unedited

Table of Contents

1. Archaeological context……………………..……………………………………………2
2. Palaeopathological examination…………………………………………………………4
2.1 ECR001……………………………………………………………………………..4
2.2 ECR003……………………………………………………………………………..9
3. Molecular pathogen screening of bulk DNA………………………………………….11
4. Authenticity of ancient *M. lepromatosis*………………………………………..…..12
5. Genome-wide comparative analyses of *M. leprae* and *M. lepromatosis*……………..14
5.1 Similarity between enrichment probes and enriched template……………………14
5.2 Genome comparisons between ancient and modern *M. lepromatosis*…………..15
5.3 Genomic comparisons against *M. lepromatosis* and related organisms…………16
5.4 Removal of regions of low complexity…………………………...………..18
6. Molecular dating………………………………………………………………………19
6.1 Model testing…………………………………………………………………19
6.2 Evaluation of strength of temporal signal…………………………………………19
6.3 Bayesian inference…………………………………………………………………..22
6.4 Sensitivity analyses………………………………………………………………22

References cited…………………………………………………………………………..29

1. Archaeological context

Oscar Eduardo Fontana Silva and José Castelleti Dellepiane

oscar.silva@museoschile.gob.cl and castelleti4mj@gmail.com

Sampling was performed on individuals from five archaeological sites in the Semi-arid region of Chile: El Cerrito, La Herradura, Peñuelas 21, Peñuelas 24, and Museo del Desierto-Conaf. All sites are located near the cities of La Serena and Coquimbo, a region characterized by low coastal mountains, valleys, and rivers that connect the coast to the Andes (Supplementary Figure 1). The first evidence of human occupation in the region dates back to the Pleistocene-Holocene transition (ca. 13,000 yBP)[1-4].

Archaeological excavations recovered 105 individuals from El Cerrito[5], 34 from Museo del Desierto-Conaf[6], 1 from La Herradura[7], 18 from Peñuelas 21, and 25 from Peñuelas 24[8-10]. These sites were inhabited by populations that relied on different subsistence strategies. While El Cerrito, La Herradura and Museo del Desierto-Conaf were cemeteries neighboring Archaic pre-ceramic fishing, hunting and gathering seasonal camps[6,7,11], Peñuelas 21 and Peñuelas 24 were cemeteries of Diaguita people, a farming and fishing culture.

In La Herradura, an ancient town located to the south of the Los Panules ravine that borders the cities of Coquimbo and La Serena, there are numerous archaeological findings that demonstrate the formation of an important geographic occupational node from archaic times in the area's mobility circuit[12]. Among them, El Cerrito is an archaeological site identified as a cemetery that was excavated in 1960, where a large number of skeletonized individuals were buried in a shell midden[13]. The bodies were distributed in two levels associated with a big diversity of artifacts of lithic and bony origin, including beads, shells, scrapers, perforators, grinding hands, and mortars, among others[5]. This site underwent radiocarbon dating, with an estimated start date of occupation ca. 3780 ± 550 yBP (Beta 6978[11]). The archaeological site of La Herradura was discovered during rescue tasks in 1998[7]. It is located close to the El Cerrito site and archaeological human remains were found along with malacological remains and a red pigment nodule.

Museo del Desierto-Conaf, Peñuelas 21 y Peñuelas 24 are archaeological sites adjacent to the Peñuelas wetland, and in the case of Museo del Desierto-Conaf, neighboring the area's drinking water. The presence of traces of inter-group violence in the Museo del Desierto skeletons is evidence of disputes over the enclave by the various groups occupying the area during the Late Archaic period[14,15].

Museo del Desierto-Conaf was excavated in 2008 and dated to the Late Archaic period, between 3,500 and 2,590 yBP. This site consists of an accumulation of shells and waste from the processing of local coastal and terrestrial resources, mainly mollusks and fish, as well as evidence of activities related to wood and leather working, and tools made of bone and lithic materials[6,14-16]. Smaller faunal deposits of marine and terrestrial mammals, such as sea lions (*Otaria flavescens*) and guanaco (*Lama guanicoe*), along with a 2.93% of rodents, mainly cururo (*Spalacopus cyanus*) and Bennett's Chinchilla rats (*Abracoma bennettii*), were also

found[14,15]. This site also presents tombs associated with camelid skeletons, with offerings of Diaguita I ceramic vessels, a tradition related to the Andean Altiplano in Tiwanaku times.

Peñuelas 21 and Peñuelas 24 correspond to cemeteries formed mostly of cysts, inside which bodies and ceramic offerings were deposited, as well as some camelid skeletons. Forty-three human skeletons and animal remains were recovered. These sites are ascribed to the Ánimas and Diaguita (Diaguita I and II) periods (ca. 1000-1540 d.C.)[8-10].

Previous paleopathological examination of some of the individuals recovered in these sites revealed, among other conditions, osteomyelitis, periostosis, *cribra orbitalia*, and a possible abscess. Although these conditions are not specific to infectious diseases, they can be associated with them[5,10]. Consequently, the aforementioned skeletal collections were selected for archaeogenetic sampling due to the presence of these possible osteological indicators of infectious disease, in an effort to maximize the probability of isolating ancient pathogen DNA.

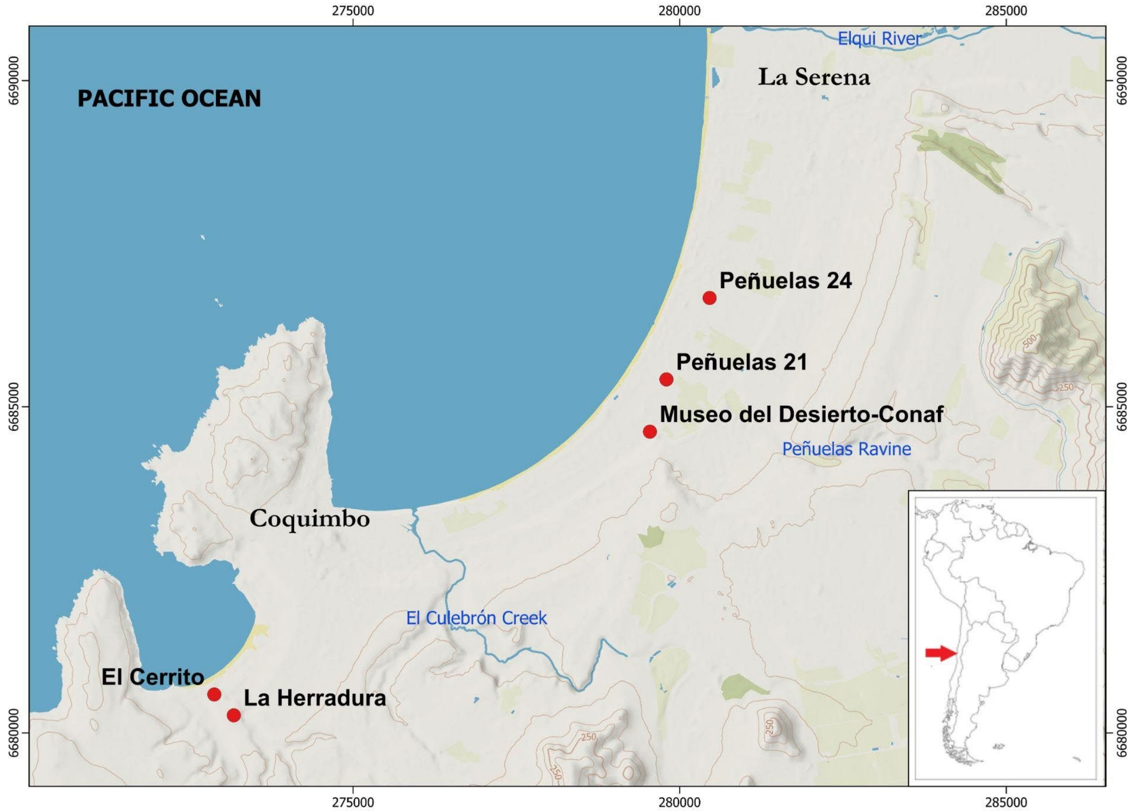

Supplementary Figure 1 – Location of the five archaeological sites from where the samples studied herein were collected. Inset: location of the region in South America. Coordinates in Universal Transverse Mercater (UTM). Map created via the MapTiler plugin within QGIS.

2. Paleopathological examination

María José Herrera-Soto and Casey L. Kirkpatrick

mariajose.herrera@uba.ar and clkirkpa@sfu.ca

The human remains included in this study were observed macroscopically under artificial lighting and the preservation of the skeletons was documented, noting the presence/absence and completeness of skeletal elements, as well as areas affected by taphonomic processes. Sex, age, and stature were then estimated using established methods[17-20]. Finally, morphological changes to the skeleton that are associated with lifestyle and/or disease were documented in detail and differentially diagnosed with reference to bioarchaeological and paleopathological manuals[17,21-26]. Here we provide a thorough description for the two individuals (ECR001 and ECR003) from whom the *M. lepromatosis* genomes were reconstructed.

2.1 Individual ECR001 (La Herradura site):

This individual has good bone preservation with >75% completeness; however, the left side of the appendicular skeleton has fewer preserved skeletal elements than the right side. The preserved bones include: the skull, vertebrae, ribs, both clavicles, both scapulae, the right humerus (fragmentary), right radius, right ulna, left *os coxa*, right femur (fragmentary), right tibia (fragmentary), right fibula (fragmentary), most of the right hand, some of the left hand, and the right calcaneus. Postmortem changes in the remains include cracking and breakage on the left parietal bone, and in the epiphyses of the upper and lower limbs. Delamination of the cortical bone is also evident in the diaphyses of the long bones and in the axial skeleton. The cranial and pelvic morphology of this individual is consistent with a male skeleton[17-27], with an estimated age of 35-39 years based on the auricular surface[18] and the cranial suture fusion stages[19]. This individual is also estimated to have a stature of 158-160 cm following De Ángel and Cisneros (2004)[20]. The following are skeletal abnormalities observed in this individual:

2.1.1. Cranium

- Inactive porosity (porotic hyperostosis stage 2[28] on the parietal and occipital bones).
- Porosity on the frontal bone in the area of the frontal sinuses, on the petrous pyramid (temporal bone), and on the palatine process.
- A lytic lesion (diameter = 3mm) on the right lateral surface of the frontal bone.
- Slightly enlarged nasal aperture (28mm width as opposed to population average width 24mm) with rounding of the margins and possible lytic reactions on the inferior surface, the anterior nasal spine, and on the maxilla below the nasal aperture.
- A lytic lesion on the antero-superior surface of the left mastoid process.
- Slight alveolar recession in the anterior teeth, though taphonomic breakage impairs complete observation.
- Advanced dental attrition.

2.1.2 Infracranial axial skeleton

- The superior and inferior surfaces of the thoracic and lumbar vertebral bodies show porosity, pitting and/or an osteolytic process along with marginal osteophytes, possibly indicating intervertebral disc disease[24].
- The T12 and lumbar vertebrae present irregularly shaped erosive lesions without sclerosis, and in the case of the T12, the lesion completely penetrates the vertebral body, though this may be postmortem damage.
- Porosity and changes to the marginal contours of the superior articular facets in the T10 and T12 vertebrae indicate osteoarthritis in these areas.
- The vertebral bodies, laminae, and spinous processes exhibit a high frequency of abnormal foramina.
- The ribs show porosity on the internal and external surfaces.

2.1.3 Appendicular skeleton

- Hand bones: The metaphyses and epiphyses of the metacarpals and phalanges show porosity, erosion and small abnormal foramina. There is also porosity and periostosis on the palmar surface of some phalanges.
- Right femur: An irregularly-shaped concave lesion with trabeculated cortical bone surrounded by a sclerotic ridge was observed on the antero-inferior surface of the right femoral neck. The morphology of this lesion suggests an Allen's fossa[29]; however, a pathological aetiology is also possible given that no other individuals in the population were similarly affected and there is additional trabeculation and porosity in areas adjacent to this lesion, abutting the growth plate, and on the femoral head. Periostosis, porosity and abnormal foramina were also observed on the distal third of the right femoral diaphysis.
- Right tibia: Mostly healed lamellar periostosis was observed in the middle and distal third of the diaphysis, which is also slightly thickened and bowed in the anteroposterior direction.
- Right fibula: Lamellar periostosis is distributed along the slightly thickened diaphysis with areas of porosity.
- Right calcaneus: There are areas of dense porosity and foramina with sclerotic bone, especially on the lateral surface.

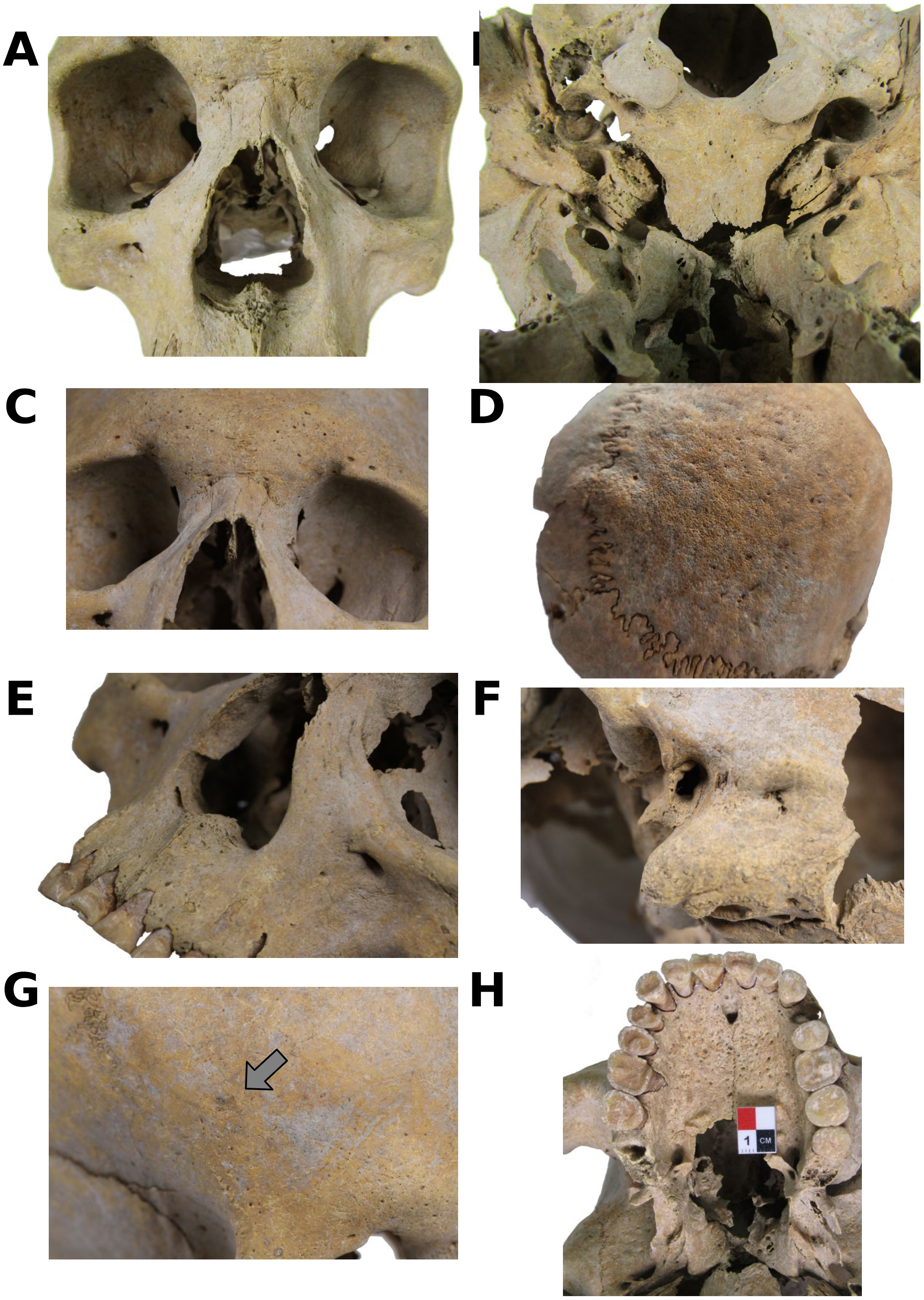

Supplementary Figure 2 – Possible pathological lesions in the skull of individual ECR001: A) Slightly enlarged nasal aperture with rounding of the margins and possible lytic reactions on the inferior surface, the anterior nasal spine, and on the maxilla below the nasal aperture, B) Porosity on the petrous pyramids of the temporal bones, C) Porosity on the superciliary arches, D) Porosity on the parietal bones, E) Rounding of the inferior margins of the nasal aperture and slight alveolar recession in the anterior teeth, F) Lytic lesion on the antero-superior surface of the left mastoid process, G) Lytic lesion (diameter = 3mm) on the right lateral surface of the frontal bone, H) Porosity on the palatine process and advanced dental attrition. Images provided by María José Herrera-Soto, Oscar Eduardo Fontana Silva, and Nicolás Pastor.

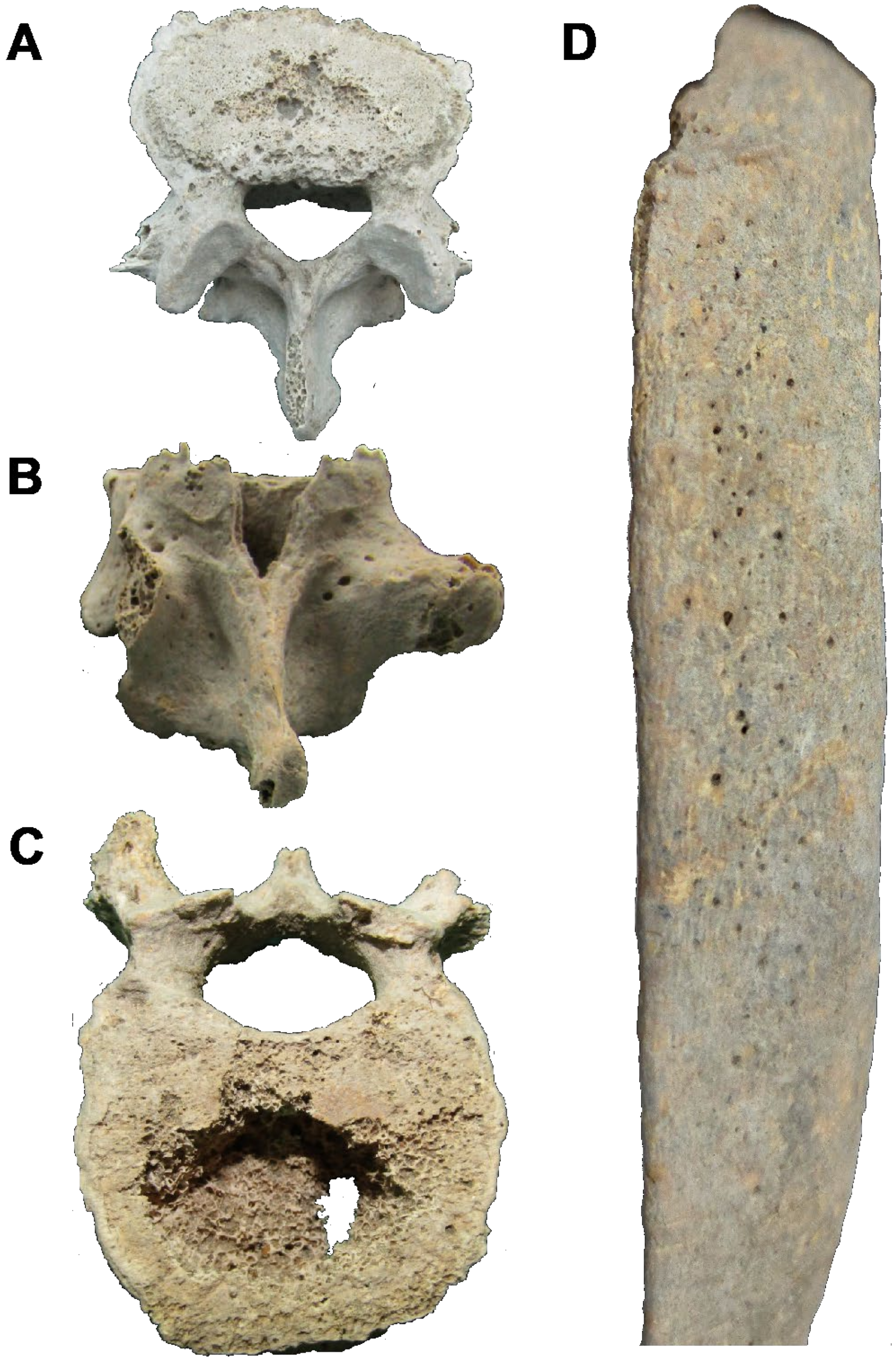

Supplementary Figure 3 – Possible pathological lesions in the axial skeleton of individual ECR001: A) Superior view of L4 vertebra with osteophytes, porosity, pitting, and osteolytic process on the vertebral body, B) Posterior view of T10 vertebra with porosity and changed contours on the superior articular facets, C) Superior view of T12 vertebra showing small osteophytes, porosity and a possible osteolytic lesion, or postmortem damage, that penetrates the whole vertebral body, D) Porosity on the external surface of a rib. Images provided by María José Herrera-Soto, Oscar Eduardo Fontana Silva, and Nicolás Pastor.

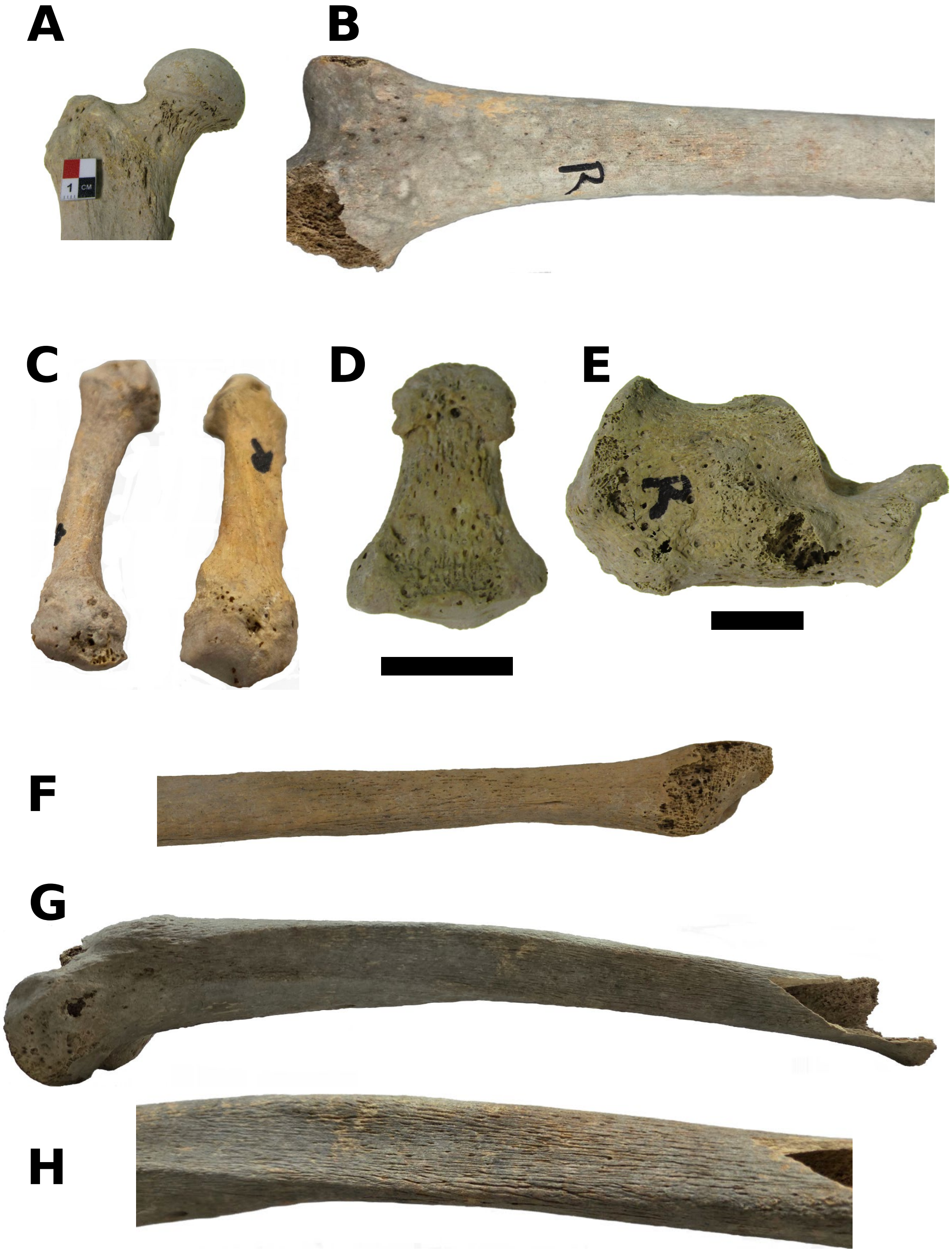

Supplementary Figure 4 – Pathological lesions in the appendicular skeleton of individual ECR001. A) Proximal right femur showing the osteolytic process (arrow) in the femoral neck; B) Periostosis, porosity and foramina in distal end of the right femur, C) Porosity and foramina in the metaphyses and epiphyses of the metacarpals, D) Porosity and periosteal new bone on a distal phalanx, E) right calcaneus showing a lesion on the peroneal tubercle that extends infero-ventrally to obscure the sulcus of the peroneus longus tendon. The large area of bone loss was caused, at least in part, by taphonomic processes; F) Right fibula with lamellar periostosis distributed along the slightly thickened diaphysis, G) Slight thickening and bowing of the right tibia, H) Right tibial shaft showing healed lamellar periostosis. Images provided by María José Herrera-Soto, Oscar Eduardo Fontana Silva, and Nicolás Pastor.

2.2 Individual ECR003 (El Cerrito site):

This individual has good bone preservation but low skeletal representation (35% of completeness). The preserved bones include: the skull (excluding the mandible), both *os coxae* (fragmentary), both femora (fragmentary), and the right tibia (fragmentary). The bones were previously treated with varnish and some bone fragments were also glued. Unfortunately, these treatments impair the observation of periosteal reactions. This individual is estimated to be biologically male[17,27] and aged 40-44 years[18] based on skeletal and dental morphology. Their stature is estimated to be 151-153 cm[20]. The following are skeletal abnormalities observed in this individual:

2.2.1. Cranium

- Slight inactive porosity (porotic hyperostosis stage 2[28]) and a bumpy, uneven surface on the parietal and occipital bones, perhaps resulting from postmortem damage or healed caries sicca, though the latter is questionable since the lesions do not affect the frontal bone and caries sicca usually spares the occipital bone.
- The right parietal exhibits a healed ovoid *antemortem* depression.
- The sagittal suture is almost completely obliterated, and 90% of the coronal and occipital sutures are obliterated. This advanced level of sutural fusion differs from the other skeletal and dental indicators of biological age, which indicate that this individual is a middle adult.
- The *pars basilaris* and the lateral and medial pterygoid plates show pitting and porosity.
- The left posterior superior alveolar foramen and the incisive foramen are widened.
- A concentration of porosity and resorptive processes is apparent lateral to the right occipital condyle.
- The right superciliary arch, in the frontal sinus area, exhibits porosity and an osteoblastic reaction. The left superciliary arch also exhibits porosity.
- The nasal foramen is slightly enlarged compared to other individuals in the population.
- There is a slight rounding of the inferior margins of the nasal aperture.
- Porosity on the palatine process.
- Moderate occlusal dental attrition.

2.2.3 Infracranial axial skeleton

- No observed pathology due to a lack of preserved skeletal elements.

2.2.4 Appendicular skeleton

- The tibia displays periostosis and slight thickening of the diaphysis and slight anteroposterior bowing.
- The tibia also has a squatting facet.

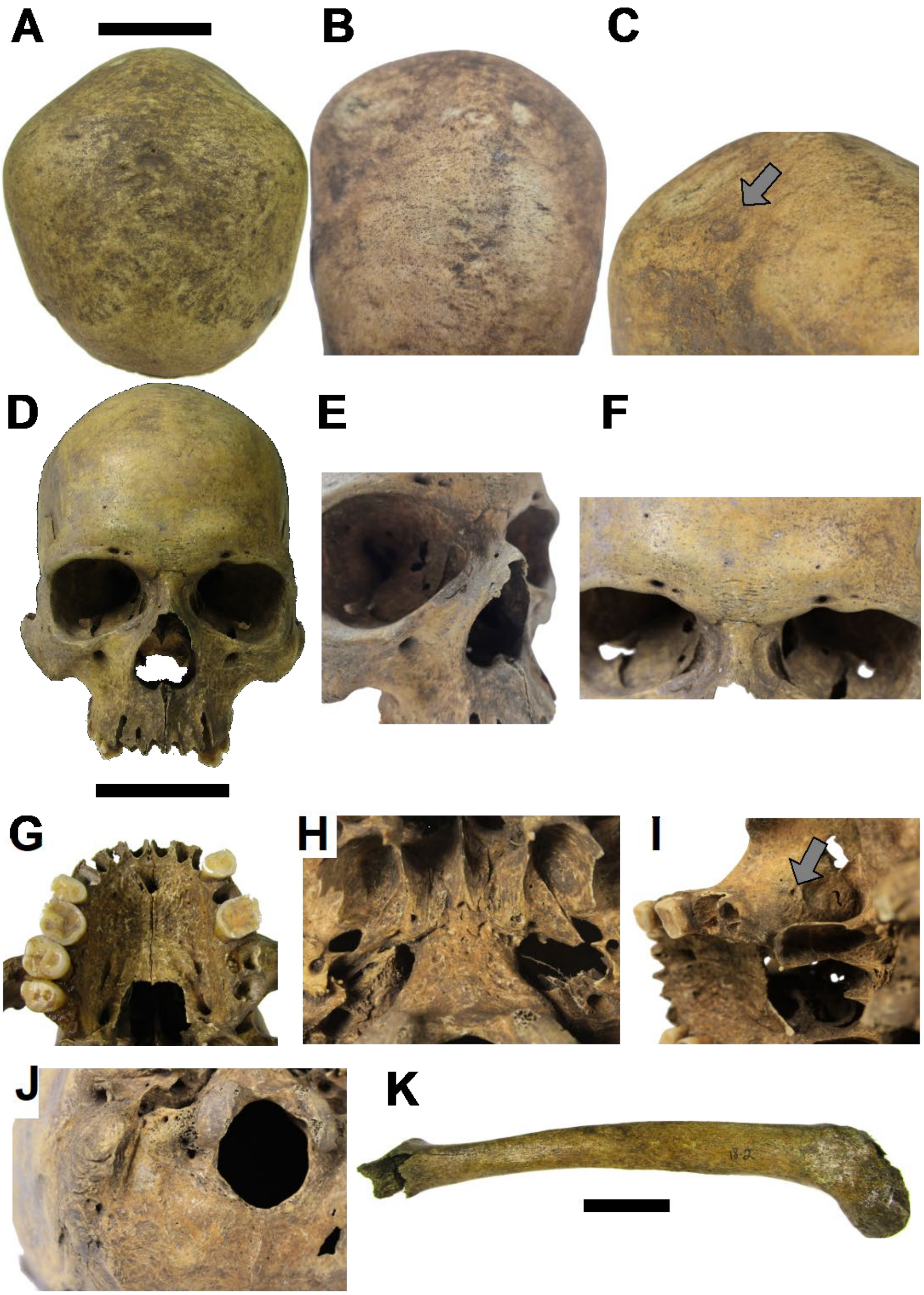

Supplementary Figure 5 – Selected skeletal elements from ECR003 A) Posterior view of cranium showing uneven parietal and occipital bones, B) Porosity on the parietal bones and near obliteration of the sagittal suture, C) Ovoid depression on right parietal bone, D) Anterior view of the skull with possible alveolar recession in anterior dentition, E) Enlarged nasal foramen and rounding of the inferior margins of the nasal aperture, F) Porosity on the superciliary arches (particularly on the right side), G) Porosity on the palatine process and moderate dental attrition, H) Pitting and porosity on the *pars basilaris* and pterygoid plates, I) Enlarged posterior superior alveolar foramen, J) Porosity and lytic lesion lateral to the right occipital condyle, K) Right tibia of individual ECR003 with slight bowing of the diaphysis and diaphyseal thickening. Images provided by María José Herrera-Soto, Oscar Eduardo Fontana Silva, and Nicolás Pastor.

3. Molecular pathogen screening of bulk DNA content

Darío A. Ramirez

darioaramirez@unc.edu.ar

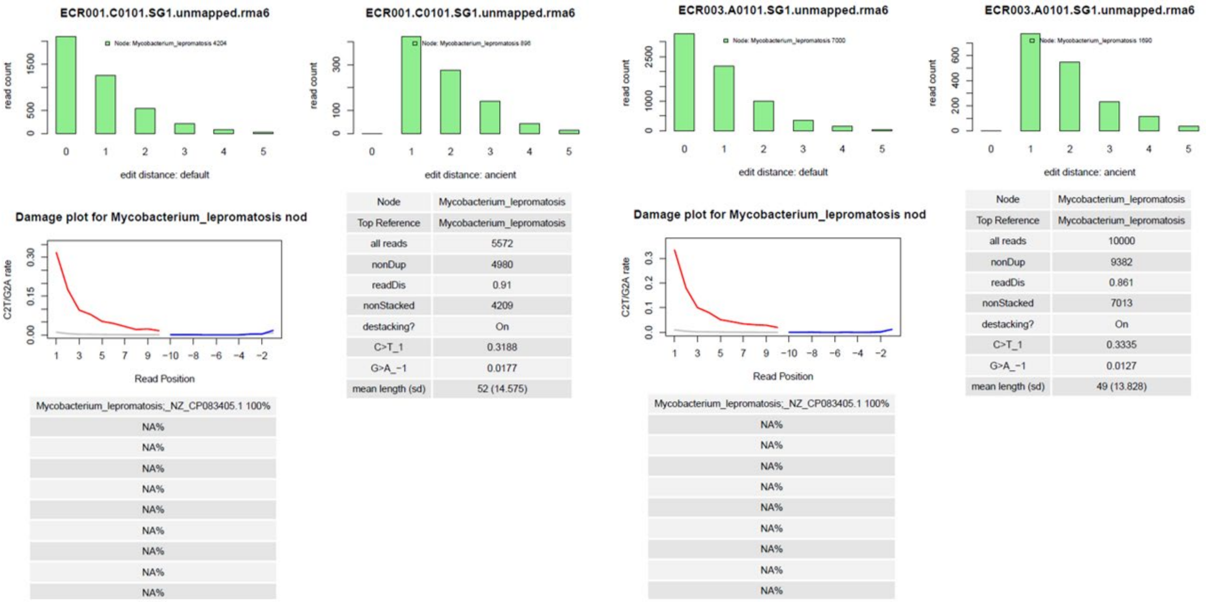

Supplementary Figure 6 – Visual results of HOPS[30] for the metagenomic screening datasets from the ECR001 and ECR003 individuals. Edit distance refers to the number of nucleotide disparities between the queried read and the reference. Read position refers to the nucleotide position in a DNA fragment counting from the 5' end.

4. Authenticity of ancient *M. lepromatosis*

Darío A. Ramirez

darioaramirez@unc.edu.ar

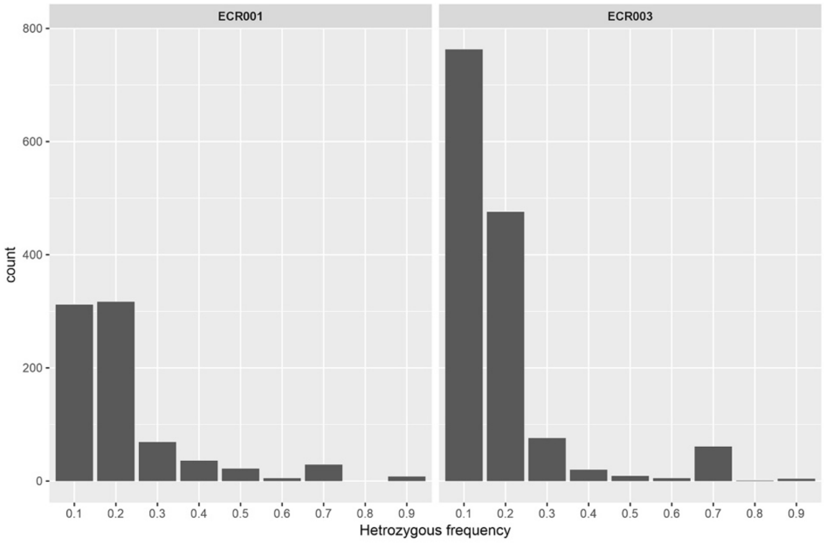

Supplementary Figure 7 – Histogram of heterozygous positions in the mapped reads for ECR001 (left) and ECR003 (right) post filtering for genomic regions of low complexity. Number of heterzygous positions (y-axis) are plotted against the frequency of the alternate allele in the reads covering the relevant position (x-axis) with a range of 0.1 (10%) to 0.9 (90%).

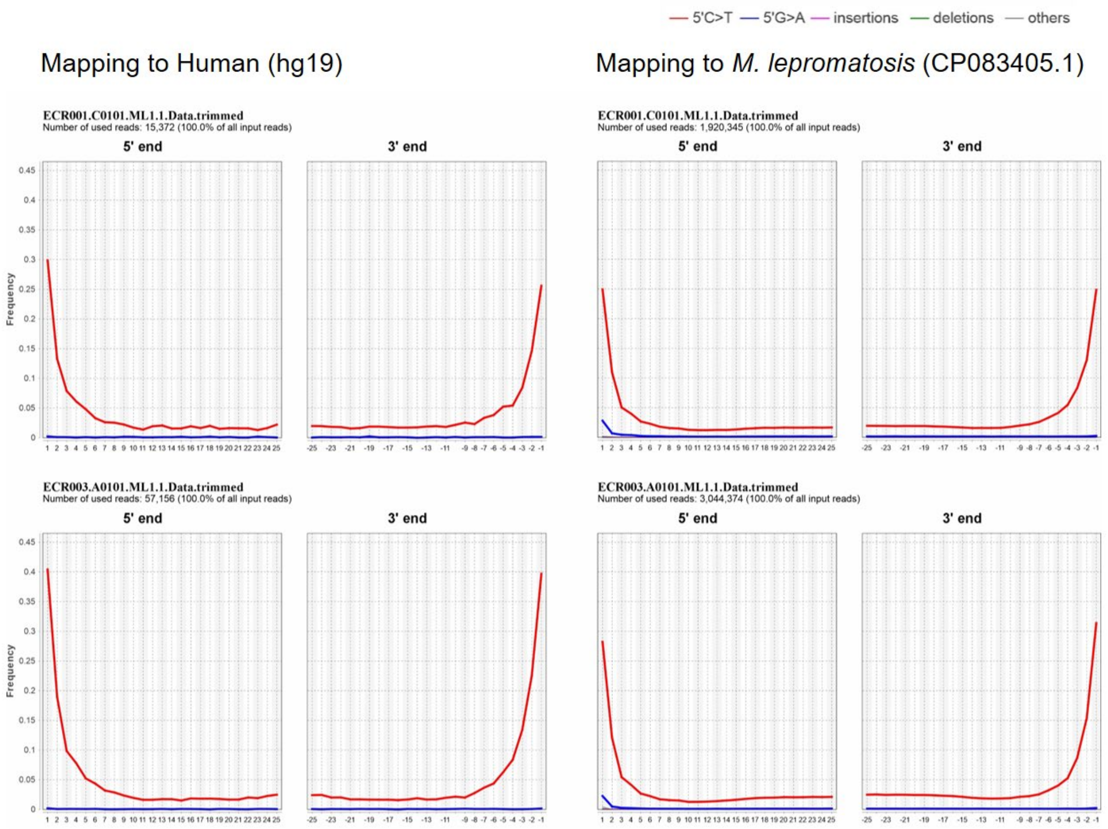

Supplementary Figure 8 – Damage plots for human and *M. lepromatosis* based on mapping of the captured data, created in DamageProfiler[31].

5. Genome-wide comparative analyses for *M. leprae* and *M. lepromatosis*

T. Lesley Sitter

lesley_sitter@eva.mpg.de

5.1 Exploration into similarity between capture probes and enriched template

Since enrichment was performed via a capture panel designed based on diversity within modern *M. leprae*, nucleotide similarity between the probes and modern *M. lepromatosis* was investigated to identify possible capture biases. This was performed via a mapping of the probe set against the *M. lepromatosis* reference with increased allowance of mismatches through iterative adjustment of the -n parameter of the bwa aln mapping software[32] implemented through the nf-core/EAGER v2.5.1 pipeline[33]. For each 52bp probe sequence, allowable mismatch was evaluated from 1 nucleotide (98% identity) up to 7 nucleotides (85% identity). Seeding was disabled.

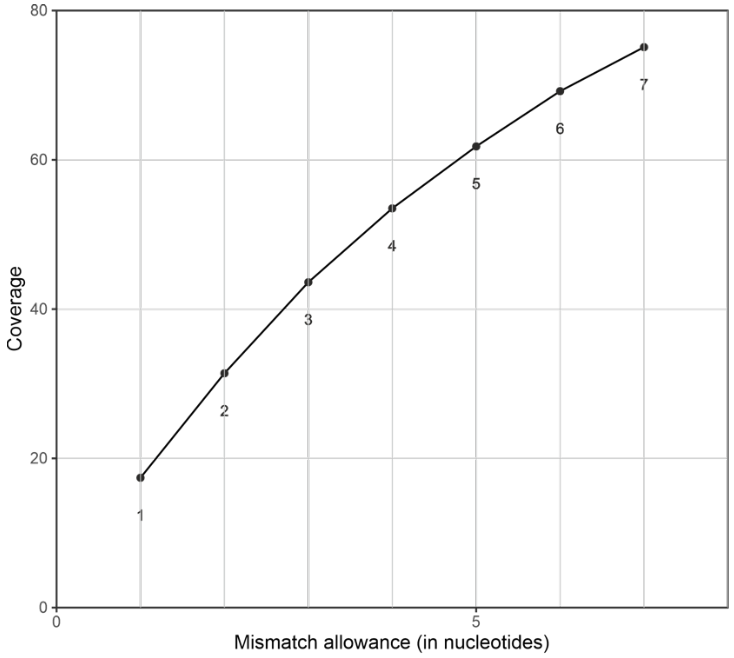

Supplementary Figure 9 – Mapping of *M. leprae* probes against the *M. lepromatosis* FJ924 reference with increasing permission of mismatches in BWA. Mismatch allowance, as specified by the -n parameter, was set to discreet integer values which specify the number of allowable mismatches in the 52bp mapping probe sequence.

These results demonstrate that enrichment of ca. 75% the genome can be expected with a mismatch rate of 7 nucleotides in a 52bp probe, or a minimum 85% identity between probe and template. Enriched datasets, however, yielded higher coverages of 82% and 88% at four-fold read support for ECR001 and ECR003, respectively with stringent mapping parameters (-n 0.1, -l 32). The increased coverage could be due to 1) mapping of reads in conserved regions from non-target sources that persist in the enriched datasets, or 2) recovery of true *M. lepromatosis* molecules that extend beyond probe-template hybridization.

To further investigate the influence of probe bias in the ancient datasets, coverage plots across annotated coding regions in the reference genome were considered (Extended Data Figure 1, Supplementary Figures 10 and 11).

5.2 – Genomic comparisons between ancient and modern M. lepromatosis

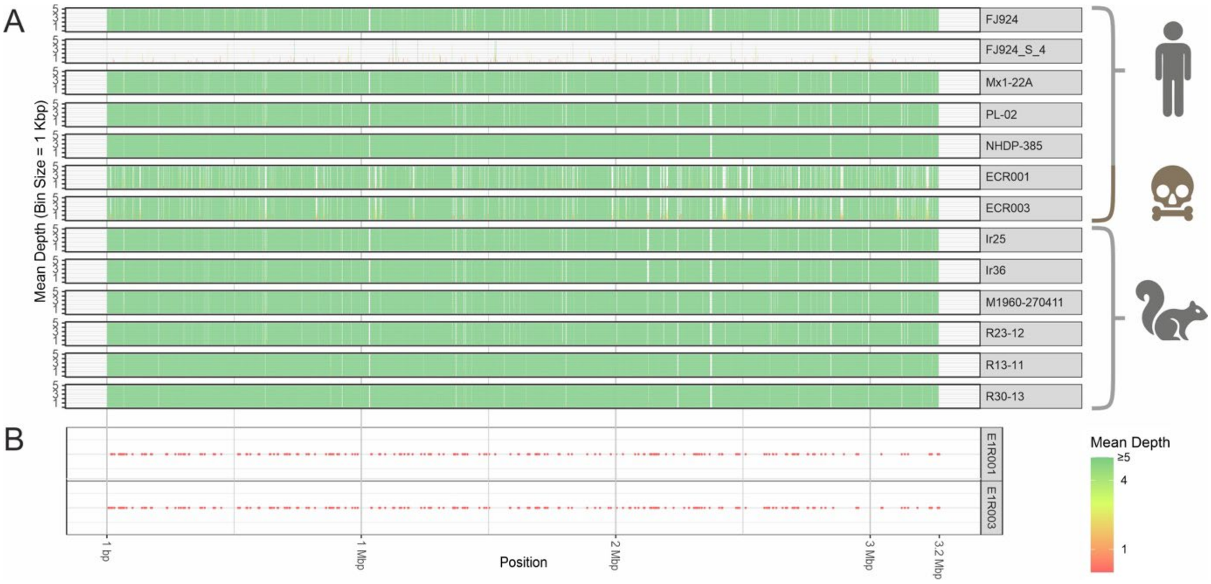

Supplementary Figure 10 – Genome plots for all datasets included in this study mapped against the FJ924 *M. lepromatosis* genome. A) Coverage plot for each sample where the average coverage is plotted in bins of 1000 bp. Values above 5x are not shown as to maintain a visually comparable depth range for all datasets. B) Graphical representation of non-reference loci identified in ECR001 and ECR003.

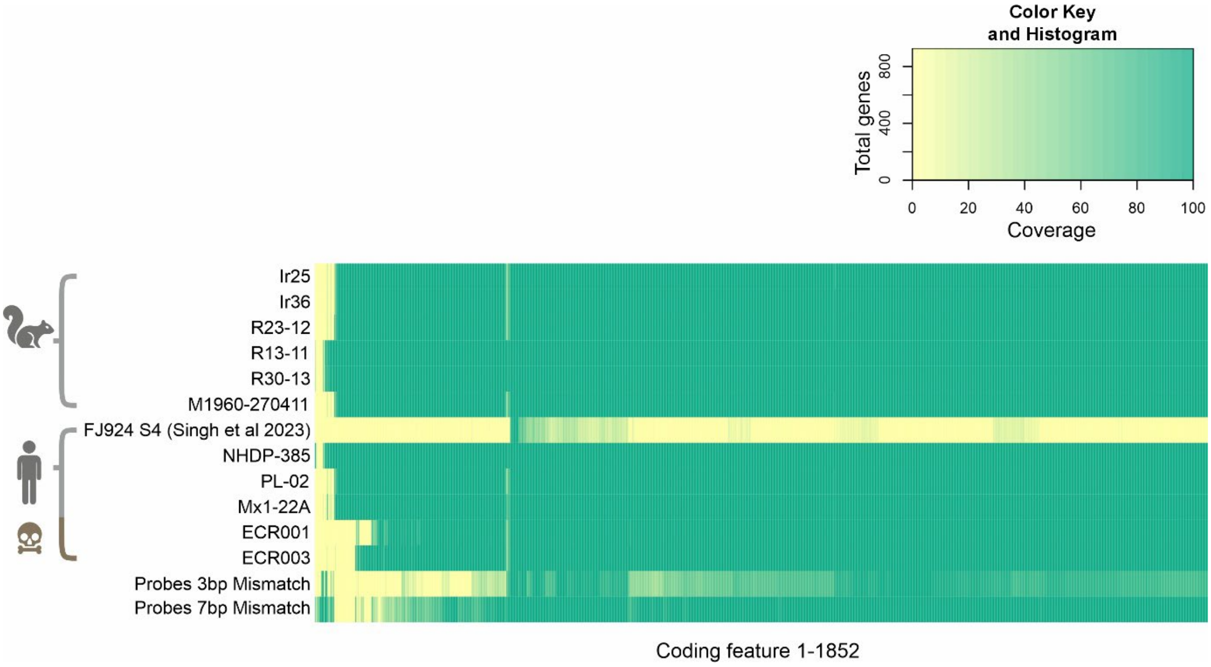

Supplementary Figure 11 – Heat map of relative mapping coverage over annotated coding regions of the FJ924 *M. lepromatosis* genome. Plot includes all 1852 annotated product-coding regions in the FJ924 reference on the y-axis. Gene labels were omitted due to illegibility.

5.3 Genomic comparisons against M. lepromatosis *and related organisms*

Genomic similarity between *M. lepromatosis* and its closest related organisms based on nucleotide identity was assessed in different ways in order to establish the position of ECR001 and ECR003 in this evolutionary trajectory. First, the four chromosomally resolved *M. leprae* genomes, along with the modern *M. lepromatosis* FJ924 reference genome, were reannotated in Prokka[34] to prevent the influence of biases in gene nomenclature. Orthology analysis of the gene features between *M. leprae* and *M. lepromatosis* was performed using Roary v3.13.0[35]. The analysis showed roughly half of the *M. lepromatosis* translated gene products to share ~50% amino acid similarity, while almost none shared 95% or more nucleotide identity suggesting a substantial divergence (Supplementary Figure 12). Using a combination of the progressiveMauve v1.1.3 tool[36] and the EasyFig BlastN visualization software[37] (Supplementary Figure 13) and LASTZ v7.0.3[38] (Supplementary Figure 14), the synteny and structural similarity of *M. lepromatosis* to the two closest related *Mycobacterium* species was assessed. This analysis revealed substantial rearrangements, reorientations, and indels. Given the similarity of ECR001 and ECR003 to *M. lepromatosis*, and the general dissimilarity between *M. lepromatosis* and its closest Mycobacterial species, it is highly probable ECR001 and ECR003 share very little genetic similarity with the common ancestor of *M. lepromatosis* and *M. leprae*.

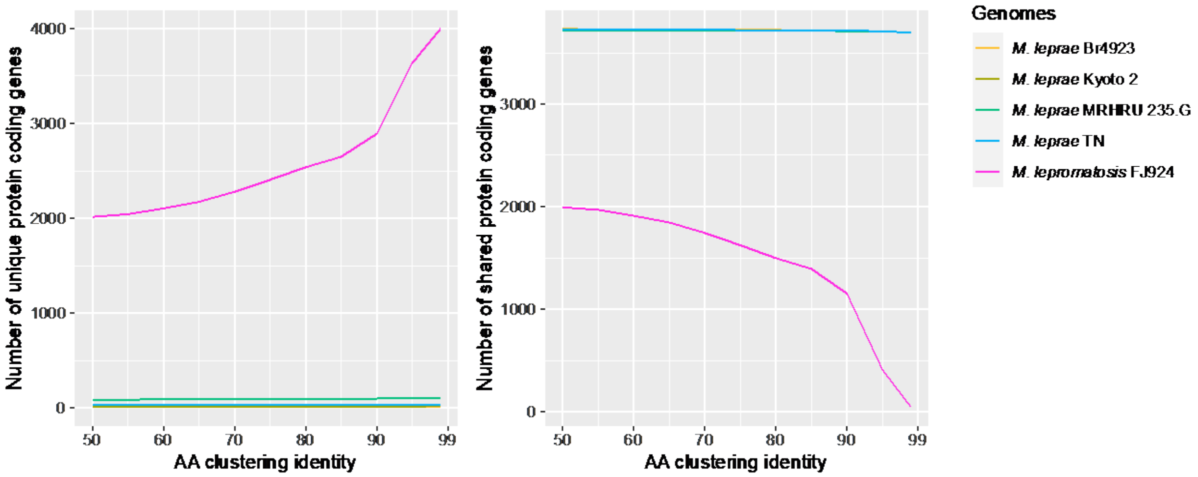

Supplementary Figure 12: Orthology analysis of the protein-coding features on the chromosomally resolved *M. lepromatosis* FJ924 genome and the *M. leprae* Br4923 (4P), Kyoto 2 (3K), MRHRU 235.G (1D) and TN (1A) reference genomes. There is no publicly available chromosomally resolved reference genome representative of the SNP genotype 2 clade. The left panel shows the number of unique features per genome plotted against amino acid identity. The right panel shows the number of features that are shared across all 5 genomes included in this analysis plotted against amino acid identity. The *M. lepromatosis* FJ924 reference genome shares roughly 50% of its protein-coding genes with those found on *M. leprae* genomes, however it lacks very few protein-coding genes that share high amino acid similarity (>95%).

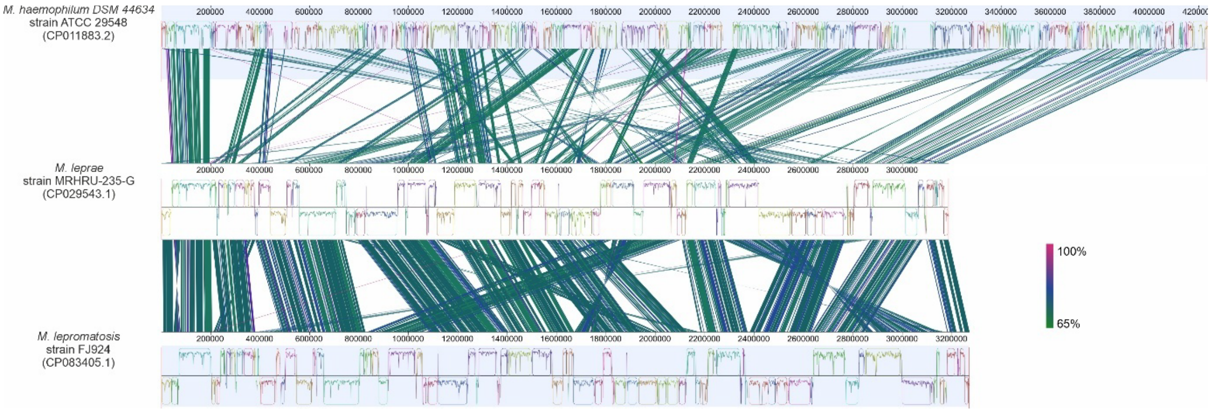

Supplementary Figure 13 – The manually combined output of the progressiveMauve and EasyFig output generated for the *M. lepromatosis* FJ924, *M. leprae* MRHRU 235.G, and *M. haemophilum* DSM 44634 strain ATCC 29548 genomes.

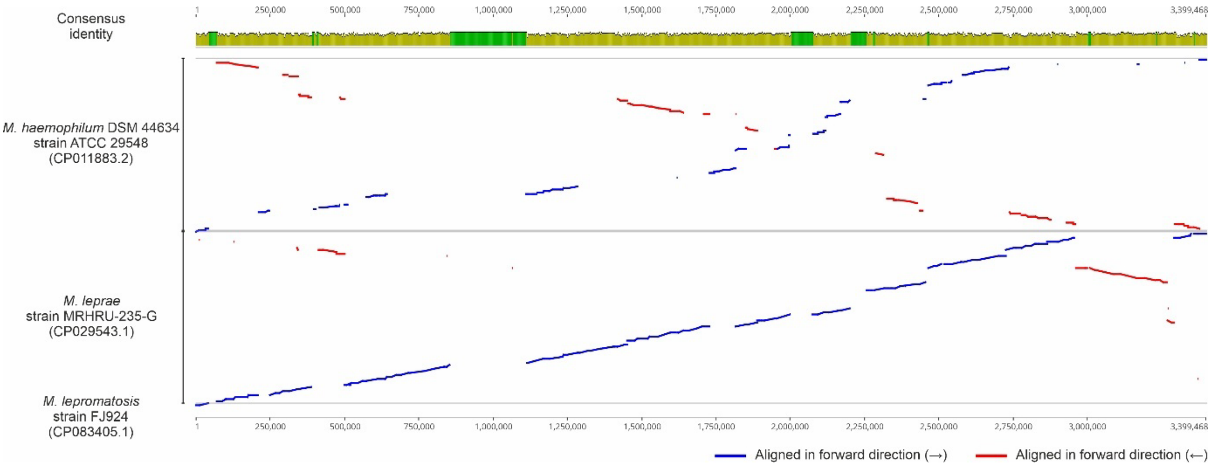

Supplementary Figure 14 - Visual representation of the LASTZ alignment between the *M. lepromatosis* FJ924, *M. leprae* MRHRU 235.G, and *M. haemophilum* DSM 44634 strain ATCC 29548 reference genomes.

5.4 – Removal of regions of low complexity for phylogenetic analysis

Several regions were filtered out prior to phylogenetic analysis. Conserved elements were manually selected from the Genbank annotated GFF file, sites prone to mapping from non-target sources were identified using SNP_Evaluation v1.0[39], recombinant regions were identified using Gubbins v3.2.1[40], and repetitive and low complexity regions were identified with DustMasker v1.0.0[41]. Recombination analysis only identified two potential recombinant regions within internal nodes, one being a 1564 bp region between position 2635870 and 2637434 and the other being a 3350bp region between position 1526069 and 1529419, which suggests that recombination occurs very rarely based on the available data. The overall filtered regions make up roughly 31,885 bp or roughly 1% of the genome. For the 16S phylogeny, ambiguous characters in the lower covered genome ECR001, which likely result from non-target mapping reads, were removed. We did not observe ambiguous characters in the higher covered genome ECR003.

6. Molecular dating

Sanni Översti
oeversti@gea.mpg.de

6.1 – Model testing

To gain deeper insights into the evolutionary history of *M. lepromatosis*, a molecular dating analysis was conducted within a Bayesian framework using the BEAST v2.7.7 software package[42]. Tip-calibrated phylogenetic trees were reconstructed from an SNP alignment comprising 650 variable sites. For the ^{14}C-dated elements ECR001 and ECR003, fixed prior estimates of 4169 yBP and 3961 yBP, respectively, were applied. For the remaining datasets, collection dates were used for tip-calibration (Supplementary Table 14).

A model averaging tool, bModelTest[43], identified the most suitable substitution model as a "three substitution types model" with equal base frequencies, i.e. the K81 model[44], which was subsequently implemented using SSM package[45] (https://zenodo.org/records/995740, last visited 13.9.2024). The most appropriate molecular clock and demographic models were inferred through the use of a nested sampling (NS) algorithm[46]. Two alternative clock models were considered – strict and optimised uncorrelated relaxed lognormal clock models[47] – along with two demographic models: the Bayesian skyline plot (BSP) model[48] and the coalescent constant population size model. Marginal likelihoods and their associated uncertainties were computed for all model combinations and compared. For each NS run, a particle count of 20 and a sub-chain length of 300,000 was employed. Comparing the ratios of marginal likelihoods using Bayes factors[49] strongly supported the BSP with a relaxed molecular clock over the other models (Supplementary Table 15A).

6.2 – Evaluation of strength in temporal signal

To resolve the absolute timescale of phylogenetic trees, reliable estimates of evolutionary rates are essential. Therefore, to evaluate whether tip-calibration alone could provide accurate results, a date-randomisation test (DRT) was performed[50]. Using the model with the highest marginal likelihood determined via nested sampling ('BSP + Relaxed clock'), twenty randomised datasets were generated with the R package TIPDATINGBEAST[51]. The performance of DRT was assessed using two criteria: the first suggests that data exhibit sufficient temporal signal only if the posterior distributions of the true and randomised datasets do not overlap[52]. The second requires that the true mean value is not included in any of the randomised posterior distributions[53]. For both parameters of interest – the clock rate and tree height – the 95% highest posterior density intervals (HPDIs) for the randomised datasets showed some overlap with the real estimates but did not overlap with the true mean values (Supplementary Figures 15 and 16). Therefore, it can be concluded that, while the data provide some evidence of temporal signal, it may not be sufficient to confidently infer evolutionary rates and timescales within a Bayesian framework.

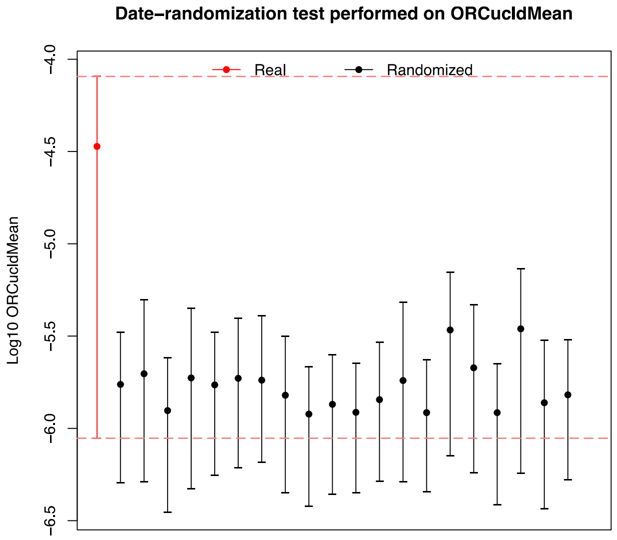

Supplementary Figure 15 **–** Date-randomisation test (DRT) conducted on the clock rate parameter of the optimised uncorrelated relaxed lognormal clock model. Date-randomisation was performed 20 times, with the real estimate shown in red and the estimates from the date-randomised datasets in black. For clarity, rate estimates are plotted on a logarithmic scale on the Y-axis. Overlapping 95% highest posterior density intervals (HPDIs) between the real and randomised estimates may suggest that the temporal signal is not strong enough to confidently infer evolutionary rates and timescales solely based on tip-dating.

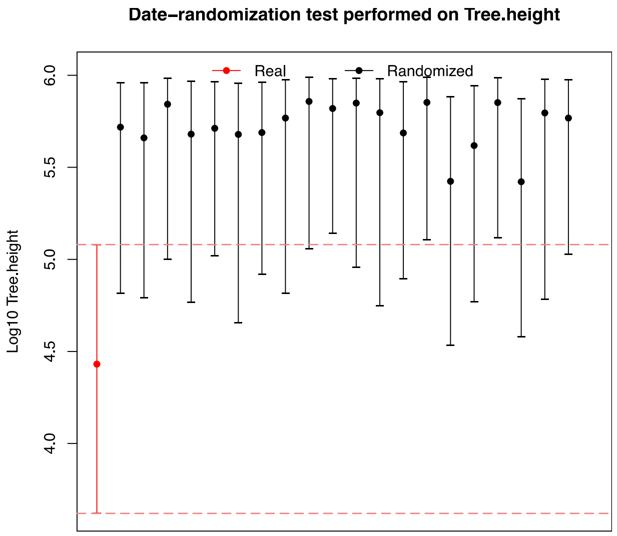

Supplementary Figure 16 – Date-randomisation test (DRT) conducted on the tree height parameter. Date-randomisation was performed 20 times, with the real estimate shown in red and the estimates from the date-randomised datasets in black. For clarity, tree height estimates are plotted on a logarithmic scale on the Y-axis. Overlapping 95% highest posterior density intervals (HPDIs) between the real and randomised estimates may suggest that the temporal signal is not strong enough to confidently infer evolutionary rates and timescales solely based on tip-dating.

6.3 Bayesian inference

Given the potential for insufficient temporal signal leading to highly biased inferences, a strict prior distribution was applied for the evolutionary rate. Since no prior estimates exist for the evolutionary rate of *M. lepromatosis*, published estimates for *M. leprae* from Schuenemann et al. (2013)[54] and Avanzi et al. (2016)[55] were used (Supplementary Table 16). Based on these estimates for the current SNP alignment of 650 sites, a normal distribution was applied with $\mu = 4.5e^{-5}$ and $\sigma = 4.5e^{-5}$ substitutions/site/year as the prior for the rate parameter. For the full *M. lepromatosis* genome, this converts to a rate with a 95% interval of normal distribution ranging from 0 to $2.70e^{-08}$ substitutions/site/year, which encompasses all previous estimates for *M. leprae*[55,56] (see also Supplementary Table 16). Unless otherwise specified, N ($4.5e^{-5}$, $4.5e^{-5}$) was used as the clock rate prior in all subsequent analyses.

For the Bayesian inference, given the relatively small sample size of 12 sequences, dimensions for BSP model parameters *bPopSize* and *bGroupSize* were set to 4. Additionally, an upper boundary of 1,000,000 was applied to *bPopSize*, and the same upper limit was set for tree height at 1,000,000 years. The MCMC chain length was set to 20,000,000 or 50,000,000 steps, depending on model complexity. Two independent MCMC chains were run for each analysis, with the first 10% of each chain discarded as burn-in. Using Tracer v1.7.2[57], we confirmed that the parallel chains converged to the same stationary distribution and that the effective sample sizes for each parameter exceeded 200. Maximum clade credibility (MCC) trees with median node heights were reconstructed using TreeAnnotator, part of the BEAST2 software package, with a 10% burn-in. MCC trees were visualised using FigTree v1.4.4 (http://tree.bio.ed.ac.uk/software/figtree/, last visited 14.9.2024).

6.4 Sensitivity analyses

Whereas the 'BSP + Relaxed clock' model showed a better fit compared to the other models, we also present the results for the main parameters of interest using the other tested models in Supplementary Table 15B and Supplementary Figures 17 and 18. Estimates for the rate parameter and tree height were consistent across all model combinations, with no statistically significant differences detected. To further assess the robustness of our results, we conducted additional sensitivity analyses. Given the limited evidence for a sufficient number of mutations accumulating over time for accurate molecular dating, we initially applied rather strict prior distribution for the clock rate parameter. To evaluate the impact of this prior, we performed two additional analyses: 1) applying a less restrictive uniform prior distribution of ($1.0e^{-10}$, 0.001) substitutions/site/year for the rate parameter, and 2) using a highly informative normal distribution with $\mu = 3.4e^{-5}$ and $\sigma = 7.0e^{-6}$ substitutions/site/year for the rate parameter. For the full *M. lepromatosis* genome the latter corresponds to a 95% interval ranging from $4.6e^{-09}$ to $9.1e^{-09}$ substitutions/site/year. This narrow prior aligns with the rate assumed by Avanzi et al. (2016)[56], which relied on *M. leprae* evolutionary rate for molecular dating of *M. lepromatosis*.

As shown in Supplementary Figures 19 and 20, the median estimates for the rate parameter and tree height are generally comparable across all prior distributions used. However, the estimates exhibit highly variable levels of uncertainty, highlighting the notable influence of prior distribution choice when the data contains limited signal. Using more permissive priors results in greater uncertainty, whereas highly informative priors, like those used in Avanzi et al.

(2016)[56], produce narrower 95% HPDIs. However, applying such highly informative priors can be problematic, especially when estimates derived from a different bacterial species are used as this approach imposes strict constraints on clock-like evolution across bacterial species that are estimated to have diverged as much as 13 million years ago[58]. This assumption generally contrasts previous findings demonstrating variation in evolutionary rates not only between closely related bacterial species but also between lineages of the same species[59]. Additionally, the choice to use such a highly informative prior for molecular dating inference without additional sensitivity analysis is further challenged by differences in the sites included in SNP alignments across studies.

Lastly, it should be noted that both demographic models employed in this study are based on coalescent theory, which in its simplest form assumes no recombination, no natural selection, and no population substructure. Since our data includes *M. lepromatosis* datasets from two different host types, the assumption of a single panmictic population is likely violated, potentially biasing parameter estimates[60,61]. While coalescent models that account for structured populations have been implemented in Bayesian framework[62], we considered the sample sizes from each host type too small to model distinct subpopulations effectively.

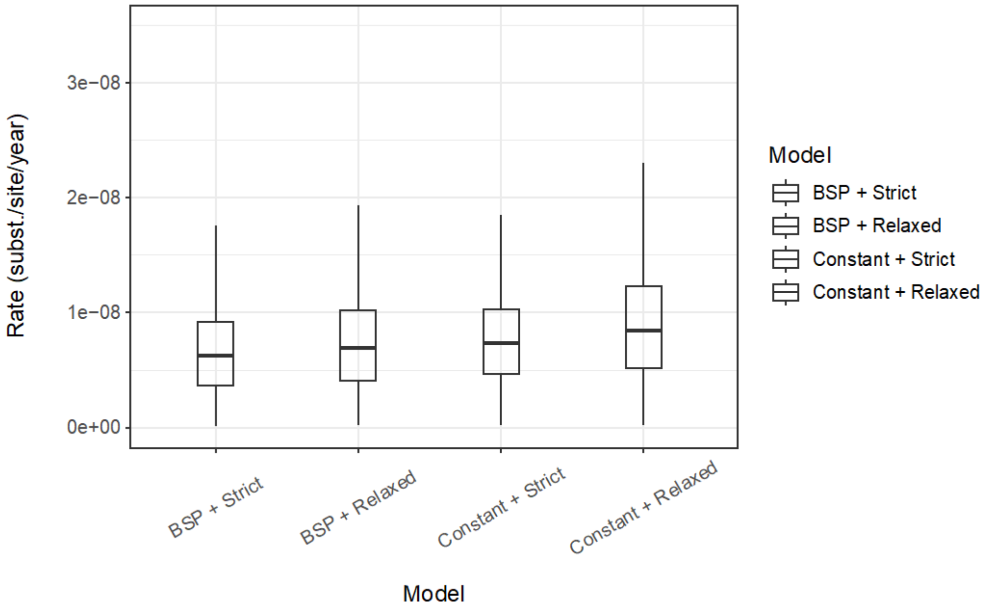

Supplementary Figure 17 – Molecular rate estimates under different model combinations. Estimates inferred using the Bayesian skyline plot (BSP) demographic model are shown in green, while those based on the coalescent constant population size model are in purple. Lighter shades represent the strict clock model, and darker shades represent the relaxed clock model. The Y-axis indicates the inferred rate estimates in substitutions/site/year extrapolated over the complete *M. lepromatosis* genome. In each boxplot, horizontal lines represent median values, with boxes spanning from the 25th to 75th percentiles. The whiskers illustrate the lower and upper 25% of values. The full posterior distribution for each model is plotted on the right side of the boxplot.

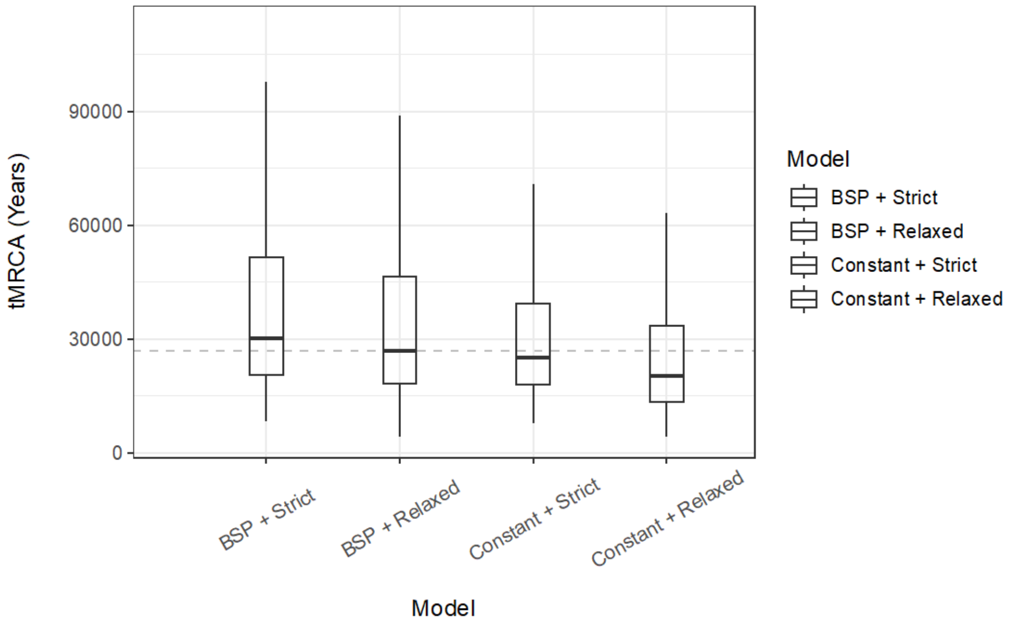

Supplementary Figure 18 – Time to most recent common ancestor (tMRCA) estimates under different model combinations. Estimates inferred using the Bayesian skyline plot (BSP) demographic model are shown in green, while those based on the coalescent constant population size model are in purple. Lighter shades represent the strict clock model, and darker shades represent the relaxed clock model. The Y-axis indicates the tMRCA estimates in years. In each boxplot, horizontal lines represent median values, with boxes spanning from the 25th to 75th percentiles. The whiskers illustrate the lower and upper 25% of values. The full posterior distribution for each model is plotted on the right side of the boxplot. The grey dashed line and shaded area represent previous tMRCA estimates from Avanzi et al. (2016)[56], with the dashed line indicating the median estimate of 26,869 years and the grey shaded area showing the 95% highest posterior density interval ([16,673; 38,458] years).

Supplementary Table 16 - Summary comparison of molecular dating analyses conducted in this study with those from Schuenemann *et al.* (2013)[55], Singh *et al.* (2015)[58], and Avanzi *et al.* (2016)[56]. Rate estimates are reported in substitutions/site/year, while divergence estimates are presented in years before present (yBP). In black, the rate estimates are extrapolated to the entire *M. leprae* or *M. lepromatosis* genome, while in grey, the rate estimates reflect the estimates calculated for each study based on the number of variable sites within the corresponding SNP alignment. For each study, we report the number of samples obtained from human and red squirrel hosts, when applicable.

	**Schuenemann et al. 2013[55]**	**Singh et al. 2015[58]**	**Avanzi et al. 2016[56]**	**This study**
Data	*M. leprae*	*M. leprae* and *M. lepromatosis*	*M. leprae* and *M. lepromatosis*	*M. lepromatosis*
Number of *M. leprae* samples	aDNA (human) x 5<br>Modern (human) x 11–12	aDNA (human) x 5<br>Modern (human) x 12	aDNA (human) x 5<br>Modern (human) x 12<br>Modern (red squirrel) x 6	–
Number of *M. lepromatosis* samples	–	Modern (human) x 1	Modern (human) x 2<br>Modern (red squirrel) x 7	aDNA (human) x 2<br>Modern (human) x 4<br>Modern (red squirrel) x 6
^{14}C calibration	*M. leprae* x 5<br><br>~1020–650 yBP	as in Schuenemann	as in Schuenemann	*M. lepromatosis* x 2<br><br>~ 4,000 yBP
Number of variable positions used	516	719,495	*M. leprae* 498<br>*M. lepromatosis* 432	650
Strength of temporal signal evaluated	Information not provided within the original publication	Information not provided within the original publication	Information not provided within the original publication	With DRT
Substitution model used	Information not provided within the original publication	Information not provided within the original publication	*M. lepra* GTR<br>*M. lepromatosis* HKY<br><br>(Original publication does not provide information on how model fit was assessed)	K81<br><br>(Best-fit model estimated with bModelTest)
Demographic model used	Information not provided within the original publication	Coalescent constant<br><br>(Original publication does not provide information on how model fit was assessed)	Coalescent constant<br><br>(Original publication does not provide information on how model fit was assessed)	Coalescent constant and Bayesian skyline plot models tested; best-fit model assessed through Nested sampling (i.e. BSP)
Molecular clock model(s) used	Strict and relaxed<br><br>(Results with both models represented and discussed)	Strict<br><br>(Original publication does not provide information on how model fit was assessed)	Strict<br><br>(Original publication does not provide information on how model fit was assessed)	Strict and relaxed clock models tested; best-fit model assessed through Nested sampling
Prior distribution used for the rate parameter	Information not provided within the original publication	Information not provided within the original publication	$6.87e^{-09}$ [$4.62e^{-09}$, $9.12e^{-09}$]	The impact of different prior distributions tested
Rate estimate inferred for	Strict clock:	–	$6.87e^{-09}$ [$4.62e^{-09}$, $9.12e^{-09}$]<br>$4.42e^{-05}$ [$2.97e^{-05}$, $5.87e^{-05}$]	–

M. leprae<br><br>(subst./site/year)	$6.13e^{-09}$ [$3.38e^{-09}$, $8.56e^{-09}$]<br>$3.81e^{-05}$ [$2.10e^{-05}$, $5.32e^{-05}$]<br><br>Relaxed clock:<br><br>$8.57e^{-09}$ [$3.61e^{-09}$, 13.2 e^{-09}]<br>$5.32e^{-05}$ [$2.24e^{-05}$, $8.87e^{-05}$]			
Rate estimate inferred for *M. lepromatosis*<br><br>(subst./site/year)	–	–	–	$6.91e^{-09}$ [$0.34e^{-09}$, $15.64e^{-09}$]<br>$3.41e^{-05}$ [$0.17e^{-05}$, $7.72e^{-05}$]<br><br>(Rates inferred with 'BSP + Relaxed clock' model, for estimates with other models, see supplementary table 15B)
Rate estimate inferred for *M. leprae* & *M. lepromatosis*<br><br>(subst./site/year)	–	$7.67e^{-09}$ [$4.2e^{-09}$, $11.1e^{-09}$]	–	
tMRCA for *M. leprae*<br><br>(Years before present)	Strict clock:<br><br>3,126 [1,975; 4,562]<br><br>Relaxed clock:<br><br>2,871 [1,350; 5,078]	3,607 [2,204; 5,525]	3,483 [2,401; 4,788]	–
tMRCA for *M. lepromatosis*<br><br>(Years before present)	–	–	26,869 [16,673; 38,458]<br><br>Human host:<br>186 [61, 328]<br><br>Red squirrel host:<br>379 [200, 695]	26,793 [4,206; 115,340]<br><br>Human host:<br>12,611 [5,304; 49,659]<br><br>Red squirrel host:<br>440 [73; 2,063]
tMRCA for *M. leprae* and *M. lepromatosis*<br><br>(Mya)	–	13.9 [8.2, 21.4]	–	

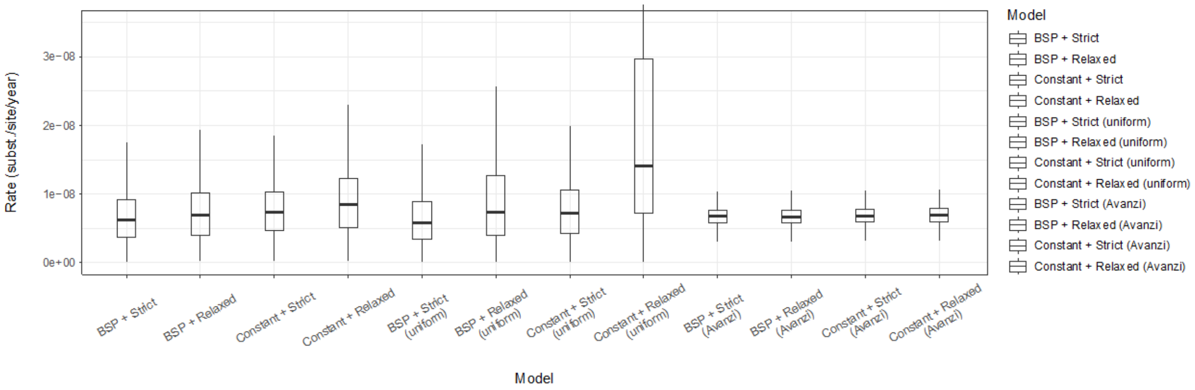

Supplementary Figure 19 – Molecular rate estimates under different model combinations and with different prior distribution on the rate parameter. For the main analyses, the estimates inferred using the Bayesian skyline plot (BSP) demographic model are shown in green, while those based on the coalescent constant population size model are in purple. Lighter shades represent the strict clock model, and darker shades represent the relaxed clock model. Estimates obtained with less stringent uniform distribution of ($1.0e^{-10}$, 0.001) are indicated with blue colour, whereas estimates obtained with highly informative prior reflecting the one used in Avanzi et al. (2016)[56] are indicated with orange colour. The Y-axis indicates the inferred rate estimates in substitutions/site/year extrapolated over the complete *M. lepromatosis* genome. In each boxplot, horizontal lines represent median values, with boxes spanning from the 25th to 75th percentiles. The whiskers illustrate the lower and upper 25% of values. The full posterior distribution for each model is plotted on the right side of the boxplot.

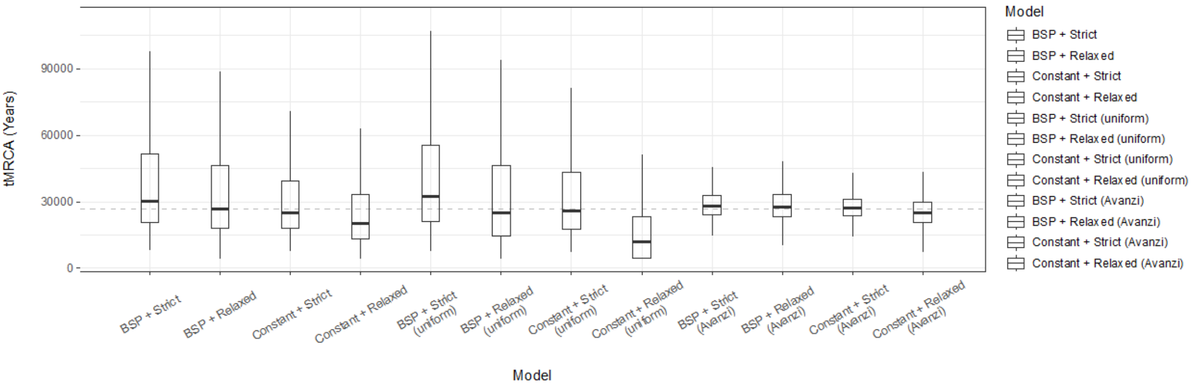

Supplementary Figure 20 **–** Time to most recent common ancestor (tMRCA) estimates under different model combinations and with different prior distributions on the rate parameter. For the main analyses, the estimates inferred using the Bayesian skyline plot (BSP) demographic model are shown in green, while those based on the coalescent constant population size model are in purple. Lighter shades represent the strict clock model, and darker shades represent the relaxed clock model. Estimates obtained with less stringent uniform distribution of (1.0e^{-10}, 0.001) are indicated with blue colour, whereas estimates obtained with highly informative prior reflecting the one used in Avanzi et al. (2016)[56] are indicated in orange colour. The Y-axis indicates the tMRCA estimates in years. In each boxplot, horizontal lines represent median values, with boxes spanning from the 25th to 75th percentiles. The whiskers illustrate the lower and upper 25% of values. The full posterior distribution for each model is plotted on the right side of the boxplot. The grey dashed line and shaded area represent previous tMRCA estimates from Avanzi et al. (2016)[56], with the dashed line indicating the median estimate of 26,869 years and the grey shaded area showing the 95% highest posterior density interval ([16,673; 38,458] years).

References

1. Jackson, D., Méndez, C. & de Souza, P. Poblamiento Paleoindio en el centro-norte de Chile: Evidencias, problemas y perspectivas de studio. *Complutum*. **15**,165-176 (2004).

2. Méndez, C. Tecnología lítica en el poblamiento pleistocénico terminal del centro de Chile. PhD thesis, Universidad Católica del Norte-Universidad de Tarapacá. (2010)

3. Méndez, C. & Jackson, D. Terminal Pleistoicene Lithic Technology and use of Space in Central Chile. *Chungara*. **47**, 53-65 (2015).

4. Núñez, L. Varela, J. & Casamiquela, R. Ocupación Paleoindia en el centro-norte de Chile: Adaptación circunlacustre en las tierras bajas. *Estudios Atacameños.* **8**,137-181 (1987).

5. Quevedo, S., Cocilovo, J., Varela, H., et al. Perfil paleodemográfico de El Cerrito (La Herradura), un grupo de pescadores arcaicos del norte semiárido de Chile *Boletín del Museo Nacional de Historia Natural.* **52**, 177-194 (2003).

6. Castelleti, J., Campano, M.A., Abarca, V., et al. Capítulo 6: El fenómeno de la violencia en sociedades cazadoras-recolectoras del semiárido chileno y su articulación al desarrollo de identidades culturales. In *Indicadores arqueológicos de violencia, guerra y conflicto en Sudamérica,* J. López Mazz and M. Berón editors, Ediciones Universitarias, Universidad de la República, Uruguay. 139-154 (2014).

7. Cobo, G. Rescate de osamenta indígena en La Herradura oriente, calle Las Flores, sector quebrada. *Ms, Museo Regional de La Serena Archive*. (1998).

8. Biskupovic, M. Excavación arqueológica en la Parcela Nº21 de Peñuelas-Coquimbo, Chile. In *Actas del IX Congreso Nacional de Arqueología Chilena.* 240-248 (1982).

9. Biskupovic, M. & Ampuero, G. Excavación arqueológica en la parcela 24 de Peñuelas, Coquimbo, Chile. In *Actas del XI Congreso Nacional de Arqueología Chilena*. Sociedad Chilena de Arqueología, Santiago de Chile. **3**:41-48 (1991).

10. Rosado, M.A. & Vernacchio-Wilson, J. Paleopathology and osteobiography of the people of Peñuelas, Chile's semiarid north. *Mem Inst Oswaldo Cruz*. **101**,85-95 (2006). doi:10.1590/s0074-02762006001000015

11. Kuzmanic I. & Castillo, G. Estadio arcaico en la costa del norte semiárido de Chile *Chungara.* **16**, 89-94 (1986).

12. Alaníz, J. Excavaciones arqueológicas en un conchal precerámico, La Herradura, provincia de Coquimbo, Chile. *Boletín del Museo Arqueológico de La Serena.* **15**,189-213 (1973).

13. Iribarren, J. Yacimientos de la Cultura del Anzuelo de Concha en el litoral de Coquimbo y Atacama. *Boletín del Museo Arqueológico de La Serena.* **11**:8-14 (1960).

14. Castelleti, J., Campano, M.A., Abarca, V., et al. Modelo de adaptación costera durante el Arcaico tardío en el semiárido: tensiones sociales y violencia interpersonal en el sitio Museo del Desierto. In *Actas XVII Congreso Nacional de Arqueología argentina.* **3**:955-959 (2010).
Facultad de Filosofía y Letras Universidad Nacional de Cuyo-Instituto de Ciencias Humanas, Sociales y Ambientales, Mendoza, Argentina

15. Castelleti, J., Biskupovic, M., Campano, M.A., et al. Adaptación costera durante el Arcaico tardío del semiárido: nuevos aportes con el estudio del sitio Museo del Desierto. In *Actas XVIII Congreso Nacional de Arqueología chilena*. In Sociedad Chilena de Arqueología, Santiago de Chile. 261-268 (2012).

16. Biskupovic, M., Fuentes, F. & Castelleti, J. Interacción costa-interior en el litoral de Coquimbo: el Caso del sitio Museo del Desierto**.** In *Tradiciones de Tierra y Mar: Antiguos pescadores, mariscadores y cazadores del semiárido,* F. Fuentes, M. Biskupovic, J. Castelleti y M. P. Retamales editors. Santiago de Chile (2010).

17. Buikstra, J. & Ubelaker, D. Chapter 3: Documentation of Sex Differences and Age Changes in Adults. In, Standards for Data Collection from Human Skeletal Remains. Arkansas Archeological Survey Research Series 44. United States of America (1994).

18. Lovejoy, C.O., Meindl, R.S., Pryzbeck, T.R. & Mensforth, R.P. Chronological metamorphosis of the auricular surface of the ilium: a new method for the determination of adult skeletal age at death. *Am J Phys Anthropol. 68*, 15-28 (1985).

19. Meindl, R.S., & Lovejoy, C.O. Ectocranial suture closure: a revised method for the determination of skeletal age at death based on the lateral-anterior sutures *Am J Phys Anthropol.* **68**:57-66 (1085).doi:10.1002/ajpa.1330680106

20. Del Angel, A, & Cisneros, H.B. Technical Note: Modification of Regression Equations Used to Estimate Stature in Mesoamerican Skeletal Remains. *Am J Physl Anthropol.* **125**:264–265 (2004).doi:10.1002/ajpa.10385

21. Hillson, S. Dental anthropology. Cambridge, UK: Cambridge University Press. 217-225 (1996).doi:10.1017/CBO9781139170697.010

22. Larsen, CS. Bioarchaeology: Interpreting behavior from the human skeleton. Second edition Cambridge University Press, Cambridge (2015).

23. Aufderheide, A. & Rodríguez-Martín. C. The Cambridge Encyclopedia of Human Paleopathology. Cambridge: Cambridge University Press (1998).

24. Waldron, T. Paleopathology. Cambridge University Press, Cambridge (2009).

25. Ortner, D.J. Identification of pathological conditions in human skeletal remains. Florida: Academic Press (2003).

26. Buikstra, J. Ortner's Identification of Pathological Conditions in Human Skeletal Remains. Third Edition. Academic Press Elsevier (2019).

27. Phenice,T.W. A Newly Developed Visual Method of Sexing the Os Pubis. *Am J of Phys Anthropol.* **30**:297-301 (1969).

28. Stuart Macadam, P. Porotic hyperostosis: Representative of a childhood condition *Am J Phys Anthropol*. **66**:391-398 (1985).

29. Göhring, A. Allen's fossa—An attempt to dissolve the confusion of different nonmetric variants on the anterior femoral neck. *International Journal of Osteoarchaeology*. **31**:513-522 (2021).

30. Hübler, R., Key, F.M., Warinner, C., et al. HOPS: automated detection and authentication of pathogen DNA in archaeological remains. *Genome Biol.* **20**, 280 (2019). https://doi.org/10.1186/s13059-019-1903-0

31. Neukamm, J., Peltzer, A. & Nieselt, K. DamageProfiler: fast damage pattern calculation for ancient DNA. *Bioinformatics*. **37**,3652–3653 (2021). doi.org/10.1093/bioinformatics/btab190

32. Li, H. & Durbin, R. Fast and accurate short read alignment with Burrows–Wheeler transform. *Bioinformatics*. **25**,1754–1760 (2009). doi.org/10.1093/bioinformatics/btp324

33. Schubert, M., Lindgreen, S., and Orlando, L. AdapterRemoval v2: rapid adapter trimming, identification, and read merging. *BMC Research Notes*. **9**,88 (2016).

34. Fellows Yates, J.A., Lamnidis, T.C., Borry, M., et al. Reproducible, portable, and efficient ancient genome reconstruction with nf-core/eager. *PeerJ.* 9:e10947 (2021). doi:10.7717/peerj.10947

35. Seeman, T. Prokka: rapid prokaryotic genome annotation. *Bioinformatics* **30**, 2068-9 (2014). doi:10.1093/bioinformatics/btu153

36. Page, A.J., Cummins, C.A., Hunt, M., et al. Roary: rapid large-scale prokaryote pan genome analysis. *Bioinformatics*. **31**,3691-3693 (2015).

37. Darling, A.E., Mau, B. & Perna, N.T. progressiveMauve: multiple genome alignment with gene gain, loss and rearrangement. *PLoS ONE*. **5**:e11147 (2010). doi.org/10.1371/journal.pone.0011147

38. Sullivan, M.J., Petty, N.K., & Beatson, S.A. Easyfig: a genome comparison visualizer. *Bioinformatics*. **27**,1009–1010 (2011). doi.org/10.1093/bioinformatics/btr039

39. Harris, R. Improved Pairwise Alignment of Genomic DNA. In *ProQuest*. (2007).

40. Keller, M., Spyrou M.A., Scheib, C.L., et al. Ancient *Yersinia pestis* genomes from across Western Europe reveal early diversification during the First Pandemic (541-750). *Proc. Nat.l Acad. Sci. USA.* **116**,12363-12372 (2019).

41. Croucher, N. J., Page, A.J., Connor, T.R., et al. Rapid phylogenetic analysis of large samples of recombinant bacterial whole genome sequences using Gubbins *Nucleic Acids Research*. **43**,e15–e15 (2015). doi.org/10.1093/nar/gku1196

42. Morgulis, A., Gertz, E.M., Schäffer, A.A., et al. A Fast and Symmetric DUST Implementation to Mask Low-Complexity DNA Sequences. *Journal of Computational Biology*. **13**,1028–1040 (2006). doi.org/10.1089/cmb.2006.13.1028

43. Bouckaert, R., Vaughn, T.G., Barido-Sottani, J., et al. BEAST 2.5: An advanced software platform for Bayesian evolutionary analysis. *PLoS Comput Biol.***15**,e1006650 (2019). doi: 10.1371/journal.pcbi.1006650

44. Bouckaert, R.R. & Drummond, A.J. bModelTest: Bayesian phylogenetic site model averaging and model comparison. *BMC Evol Biol.* **17**,1–11 (2017). doi:10.1186/s12862-017-0890-6

45. Kimura, M. Estimation of evolutionary distances between homologous nucleotide sequences. *Proc Natl Acad Sci*. **78**,454–458 (1981). doi: 10.1073/pnas.78.1.454

46. Boukaert, R., Xie, D. BEAST2-Dev/substmodels: Standard Nucleotide Substitution Models v1.0.1 (v1.0.1). Zenodo. doi.org/10.5281/zenodo.995740

47. Russel, P.M., Brewer, B.J., Klaere, S., et al. Model Selection and Parameter Inference in Phylogenetics Using Nested Sampling. *Syst Biol*. **68**,219–233 (2019). doi: 10.1093/sysbio/syy050

48. Douglas, J., Zhang, R. & Bouckaert, R. Adaptive dating and fast proposals: Revisiting the phylogenetic relaxed clock model. *PLOS Comput Biol.***17**,e1008322 (2021). doi:10.1371/journal.pcbi.1008322

49. Drummond, A.J., Rambaut, A., Shapiro, B., • et al. Bayesian Coalescent Inference of Past Population Dynamics from Molecular Sequences. *Mol Biol Evol*. **22**, 1185–1192 (2005). doi:10.1093/molbev/msi103

50. Kass, R.E. & Raftery, A.E. Bayes factors. *J Am Stat Assoc.* **90**, 773–795 (1995). doi:10.1080/01621459.1995.10476572

51. Ramsden, C., Melo, F.L., Figueiredo, L.M., et al. High Rates of Molecular Evolution in Hantaviruses. *Mol Biol Evol*. **25**,1488–1492 (2008). doi:10.1093/molbev/msn093

52. Rieux, A. & Khatchikian, C.E. 2017. tipdatingbeast: an r package to assist the implementation of phylogenetic tip-dating tests using beast. *Mol Ecol Resour.* **17**,608–613 (2017). doi: 10.1111/1755-0998.12603

53. Ramsden, C., Holmes, E.C. & Charleston, M.A. Hantavirus Evolution in Relation to Its Rodent and Insectivore Hosts: No Evidence for Codivergence. *Mol Biol Evol*. **26**, 143–153 (2009). doi: 10.1093/molbev/msn234

54. Firth, C., Kitchen, A., Shaprio, B., et al. Using Time-Structured Data to Estimate Evolutionary Rates of Double-Stranded DNA Viruses. *Mol Biol Evol.* **27**,2038–2051 (2010). doi: 10.1093/molbev/msq088

55. Schuenemann, V.J., Singh, P., Mendum, T.A., et al. Genome-Wide Comparison of Medieval and Modern *Mycobacterium leprae*. *Science*. **341**, 179–183 (2013). doi:10.1126/science.1238286

56. Avanzi, C., Del-Pozo, J., Benjak, A., et al. Red squirrels in the British Isles are infected with leprosy bacilli. *Science*. **354**,744–747 (2016). doi:10.1126/science.aah3783

57. Rambaut, A., Drummond, A.J., Xie, D., • et al. Posterior summarization in Bayesian phylogenetics using Tracer 1.7. *Syst Biol.* **67**,901–904 (2018). doi:10.1093/sysbio/syy032

58. Singh, P., Benjack, A., Schuenemann, V.J., et al. Insight into the evolution and origin of leprosy bacilli from the genome sequence of *Mycobacterium lepromatosis*. *Proc Natl Acad Sci.* **112**,4459–4464 (2015). doi:10.1073/pnas.1421504112

59. Duchene et al. (2016) Genome-scale rates of evolutionary change in bacteria. *Microbial genomics*, *2*(11), e000094. doi.org/10.1099/mgen.0.000094

60. Navascués, M. & Emerson, B.C. Elevated substitution rate estimates from ancient DNA: model violation and bias of Bayesian methods. *Mol Ecol.* **18**,4390–4397 (2009). doi:10.1111/j.1365-294X.2009.04333.x

61. Heller, R., Chikhi, L. & Siegismund, H.R. The Confounding Effect of Population Structure on Bayesian Skyline Plot Inferences of Demographic History. *PLoS ONE*. 8,e62992 (2013).doi:10.1371/journal.pone.0062992

62. Vaughan, T.G., Kühnert, D., Popinga, A., et al. Efficient Bayesian inference under the structured coalescent. *Bioinformatics*. **30**,2272–2279 (2014). doi:10.1093/bioinformatics/btu201
